# Supplementary material for: Influences of aging and mating history in males on paternity success in the red flour beetle Tribolium castaneum
Source: PLoS One. 2024 Dec 23;19(12):e0316008. doi: 10.1371/journal.pone.0316008 (PMC11665990; doi:10.1371/journal.pone.0316008)
Supplement: S1 Fig — (DOCX) [file pone.0316008.s001.docx]

**Supplementary information**


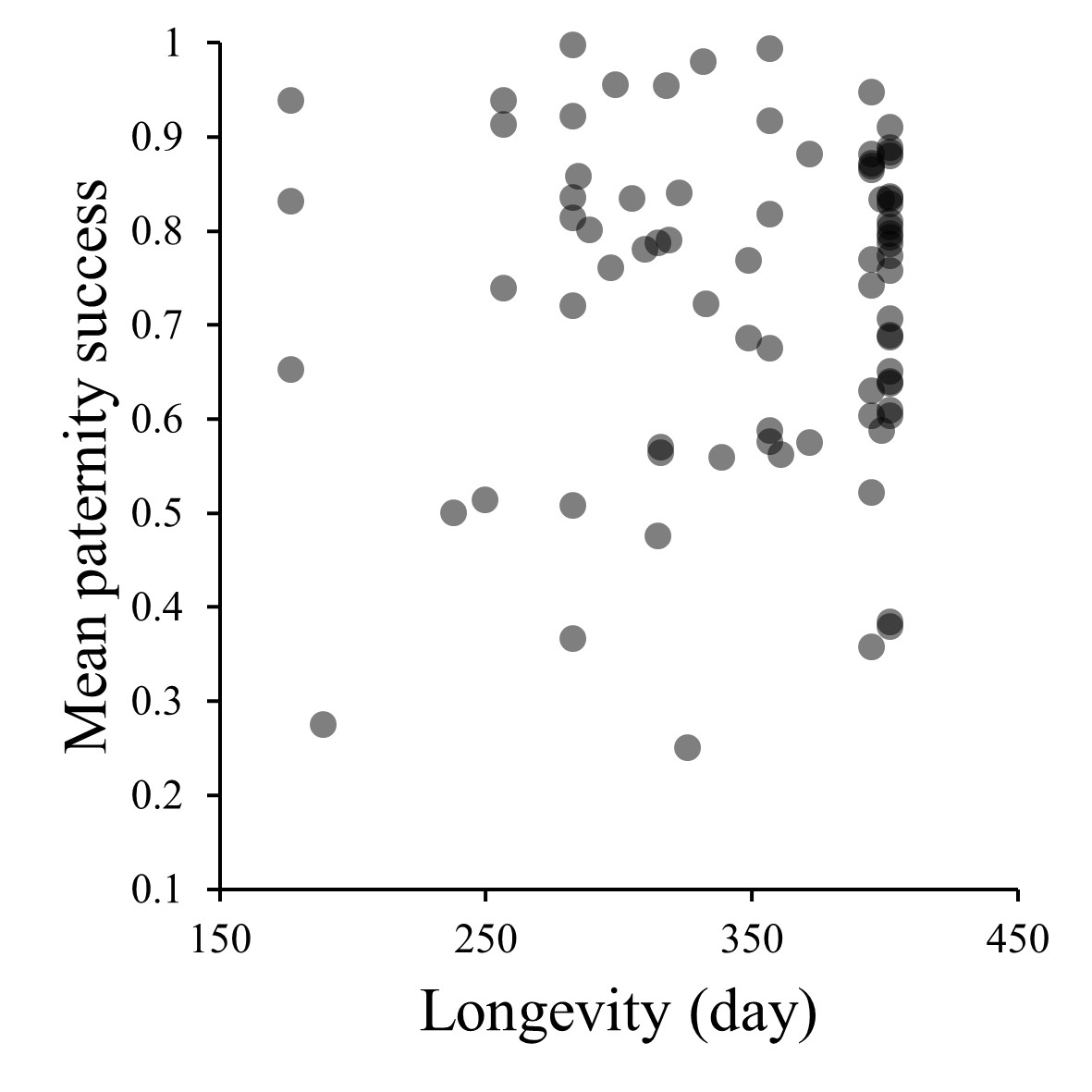


**Figure S1**. Relationship between longevity and their paternity success (mean).
