# Supplementary material for: Influences of aging and mating history in males on paternity success in the red flour beetle Tribolium castaneum
Source: PLoS One. 2024 Dec 23;19(12):e0316008. doi: 10.1371/journal.pone.0316008 (PMC11665990; doi:10.1371/journal.pone.0316008)

PLOS ONE

Influences of aging and mating history in males on their paternity in the red flour beetle Tribolium castaneum

--Manuscript Draft--

**Manuscript Number:**	PONE-D-24-05330
**Article Type:**	Research Article
**Full Title:**	Influences of aging and mating history in males on their paternity in the red flour beetle Tribolium castaneum
**Short Title:**	Influences of aging and mating history on their paternity
**Corresponding Author:**	Kentarou Matsumura<br>Graduate School of Environmental, Life, Nature Science<br>Okayama, JAPAN
**Keywords:**	sexual selection; sperm competition; aging; Tribolium castaneum
**Abstract:**	The investment in reproductive traits by male is often associated with their age. For example, several previous studies have reported that older males increase their investment in current reproduction than younger males. However, sperm quality often declines with male aging, and the interaction between aging and mating history in males may also affect to post-copulatory sexual selection. However, relationship between male aging and paternity remains unclear. In this study, we investigated paternity success of males at each age in the red flour beetle Tribolium castaneum. To investigate effects of mating history, we also compared the paternity success between older males who experienced some copulations (mated male) and older males who did not experience copulation (naive male). In our results, we found that paternity success decreased with age, regardless of male mating history. Furthermore, naive old males showed significantly higher paternity success than mated old males. This result suggests that naive older males increased their current investment in reproduction. Our study suggests that interaction between aging and mating history in males is affected to paternity success in T. castaneum.
**Order of Authors:**	Kentarou Matsumura
	Renya Kawakami
**Additional Information:**	
**Question**	**Response**
**Financial Disclosure**<br><br>Enter a financial disclosure statement that describes the sources of funding for the work included in this submission. Review the submission guidelines for detailed requirements. View published research articles from *PLOS ONE* for specific examples.<br><br>This statement is required for submission and **will appear in the published article** if the submission is accepted. Please make sure it is accurate.	Yes

Powered by Editorial Manager® and ProduXion Manager® from Aries Systems Corporation

**Funded studies**<br>Enter a statement with the following details:<br>• Initials of the authors who received each award<br>• Grant numbers awarded to each author<br>• The full name of each funder<br>• URL of each funder website<br>• Did the sponsors or funders play any role in the study design, data collection and analysis, decision to publish, or preparation of the manuscript?<br><br>Did you receive funding for this work?	
Please add funding details.<br>as follow-up to "**Financial Disclosure**<br><br>Enter a financial disclosure statement that describes the sources of funding for the work included in this submission. Review the submission guidelines for detailed requirements. View published research articles from *PLOS ONE* for specific examples.<br><br>This statement is required for submission and **will appear in the published article** if the submission is accepted. Please make sure it is accurate.<br><br>**Funded studies**<br>Enter a statement with the following details:<br>• Initials of the authors who received each award<br>• Grant numbers awarded to each author<br>• The full name of each funder<br>• URL of each funder website<br>• Did the sponsors or funders play any role in the study design, data collection and analysis, decision to publish, or preparation of the manuscript?<br><br>Did you receive funding for this work?"	This work was supported by JSPS KAKENHI Grant Numbers 22K20664 and 23K14264 to K.M.
Please select the country of your main research funder (please select carefully as in some cases this is used in fee calculation).	JAPAN - JP

as follow-up to "**Financial Disclosure**<br><br>Enter a financial disclosure statement that describes the sources of funding for the work included in this submission. Review the submission guidelines for detailed requirements. View published research articles from *PLOS ONE* for specific examples.<br><br>This statement is required for submission and **will appear in the published article** if the submission is accepted. Please make sure it is accurate.<br><br>**Funded studies**<br>Enter a statement with the following details:<br>• Initials of the authors who received each award<br>• Grant numbers awarded to each author<br>• The full name of each funder<br>• URL of each funder website<br>• Did the sponsors or funders play any role in the study design, data collection and analysis, decision to publish, or preparation of the manuscript?<br><br>Did you receive funding for this work?"	
**Competing Interests**<br><br>Use the instructions below to enter a competing interest statement for this submission. On behalf of all authors, disclose any competing interests that could be perceived to bias this work—acknowledging all financial support and any other relevant financial or non-financial competing interests.<br><br>This statement is required for submission and **will appear in the published article** if the submission is accepted. Please make sure it is accurate and that any funding sources listed in your Funding Information later in the submission form are also declared in your Financial Disclosure statement.	The authors have declared that no competing interests exist.

Powered by Editorial Manager® and ProduXion Manager® from Aries Systems Corporation

View published research articles from *PLOS ONE* for specific examples.<br><br>**NO authors have competing interests**<br><br>Enter: *The authors have declared that no competing interests exist.*<br><br>**Authors with competing interests**<br><br>Enter competing interest details beginning with this statement:<br><br>*I have read the journal's policy and the authors of this manuscript have the following competing interests: [insert competing interests here]*<br><br>* typeset	
**Ethics Statement**<br><br>Enter an ethics statement for this submission. This statement is required if the study involved:<br><br>• Human participants<br>• Human specimens or tissue<br>• Vertebrate animals or cephalopods<br>• Vertebrate embryos or tissues<br>• Field research<br><br>Write "N/A" if the submission does not require an ethics statement.<br><br>General guidance is provided below. Consult the submission guidelines for detailed instructions. **Make sure that all information entered here is included in the Methods section of the manuscript.**	N/A

**Format for specific study types**<br><br>**Human Subject Research (involving human participants and/or tissue)**<br>• Give the name of the institutional review board or ethics committee that approved the study<br>• Include the approval number and/or a statement indicating approval of this research<br>• Indicate the form of consent obtained (written/oral) or the reason that consent was not obtained (e.g. the data were analyzed anonymously)<br><br>**Animal Research (involving vertebrate animals, embryos or tissues)**<br>• Provide the name of the Institutional Animal Care and Use Committee (IACUC) or other relevant ethics board that reviewed the study protocol, and indicate whether they approved this research or granted a formal waiver of ethical approval<br>• Include an approval number if one was obtained<br>• If the study involved *non-human primates*, add *additional details* about animal welfare and steps taken to ameliorate suffering<br>• If anesthesia, euthanasia, or any kind of animal sacrifice is part of the study, include briefly which substances and/or methods were applied<br><br>**Field Research**<br><br>Include the following details if this study involves the collection of plant, animal, or other materials from a natural setting:<br>• Field permit number<br>• Name of the institution or relevant body that granted permission	
**Data Availability**<br><br>Authors are required to make all data underlying the findings described fully available, without restriction, and from the time of publication. PLOS allows rare exceptions to address legal and ethical concerns. See the PLOS Data Policy and FAQ for detailed information.	No - some restrictions will apply

A Data Availability Statement describing where the data can be found is required at submission. Your answers to this question constitute the Data Availability Statement and **will be published in the article**, if accepted.<br><br>**Important:** Stating 'data available on request from the author' is not sufficient. If your data are only available upon request, select 'No' for the first question and explain your exceptional situation in the text box.<br><br>Do the authors confirm that all data underlying the findings described in their manuscript are fully available without restriction?	
**Describe where the data may be found in full sentences. If you are copying our sample text, replace any instances of XXX with the appropriate details.**<br><br>• If the data are **held or will be held in a public repository**, include URLs, accession numbers or DOIs. If this information will only be available after acceptance, indicate this by ticking the box below. For example: *All XXX files are available from the XXX database (accession number(s) XXX, XXX.).*<br>• If the data are all contained **within the manuscript and/or Supporting Information files**, enter the following: *All relevant data are within the manuscript and its Supporting Information files.*<br>• If neither of these applies but you are able to provide **details of access elsewhere**, with or without limitations, please do so. For example:<br><br>*Data cannot be shared publicly because of [XXX]. Data are available from the XXX Institutional Data Access / Ethics Committee (contact via XXX) for researchers who meet the criteria for access to confidential data.*<br><br>*The data underlying the results presented in the study are available from (include the name of the third party*	data available on request from the author

and contact information or URL).<br>• This text is appropriate if the data are owned by a third party and authors do not have permission to share the data.<br><br>* typeset	
Additional data availability information:	

Click here to access/download;Manuscript;Manuscript_20240208_KM.docx

Full title

Influences of aging and mating history in males on their paternity in the red flour beetle *Tribolium castaneum*

Short title

Influences of aging and mating history on their paternity

Authors

Renya Kawakami1

Kentarou Matsumura2*

1. Graduate School of Agriculture, Kagawa University, Kagawa, Japan
2. Graduate School of Environmental, Life, Nature Science and Technology, Okayama University, Okayama, Japan

*Corresponding author

E-mail: ag20110@s.okayama-u.ac.jp (KM)

Abstract

The investment in reproductive traits by male is often associated with their age. For example, several previous studies have reported that older males increase their investment in current reproduction than younger males. However, sperm quality often declines with male aging, and the interaction between aging and mating history in males may also affect to post-copulatory sexual selection. However,

relationship between male aging and paternity remains unclear. In this study, we investigated paternity success of males at each age in the red flour beetle *Tribolium castaneum*. To investigate effects of mating history, we also compared the paternity success between older males who experienced some copulations (mated male) and older males who did not experience copulation (naive male). In our results, ~~we found~~ that paternity success decreased with age, regardless of male mating history. Furthermore, naive old males showed significantly higher paternity success than mated old males. This result suggests that naive older males increased their current investment in reproduction. Our study suggests that interaction between aging and mating history in males ~~is~~ affected ~~to~~ paternity success in *T. castaneum*.

Keywords: sexual selection, sperm competition, aging, *Tribolium castaneum*

Introduction

Sexual selection is the selection pressure that induces the evolution of traits which leads to increased reproductive success [1, 2]. For example, males often have exaggerated weapon traits that are used in male–male combat for mating opportunities with females. Moreover, males often have gaudy ornamental traits and show unique courtship behaviour to get the female to accept mating. Since this occurs before copulation, these processes are called “premating sexual selection.” Conversely, in polyandrous species, male sperm is not necessarily used during fertilization. Then, male–male competition also occurs for egg fertilization inside the female, and is called “sperm competition.” The sperm competition leads to the evolution of traits that are beneficial in this competition. In post-copulatory

sexual selection, not only sperm competition as male-male competition, "cryptic female choice," in which the female choice for sperm in the body after mating, is also affects male paternity [3-5]. In paternity, however, it is very difficult to separate the effects of CFC from those of sperm competition [5]. Therefore, although males need to invest resources for reproduction both pre- and post-copulation, the resource allocation to reproduction by males is expected to vary depending on various factors, and clarifying these factors is important for understanding sexual selection.

One factor affecting male investment in reproductive traits is male aging. Males are often injured or death in intense male-male combat. In young males, it may be more adaptive to avoid participating in intense male combat because of the many future reproductive opportunities. Conversely, older males may increase their investment in male-male combat more than younger males because they are not expected to have increased reproductive opportunities in the future [6-8]. Thus, males should restrain investment in reproduction during early life (young) and increase it during later life (old) when they have few chances of future reproduction [7]. It is predicted that young males decrease investment in present reproduction to invest in the future, whereas old males invest in present reproduction and not in the future. In a theoretical study of evolutionarily stable strategy (ESS) of age dependent sexual advertisement [9], it was revealed that in many cases where sexual trait values were increased with aging were ESS. Indeed, in the broad-horned flour beetle *Gnatocerus cornutus*, older male fights harder than younger males in male–male combat for females [10], and the old male butterfly *Hypolimnas bolina* fights longer than young males [8]. Moreover, the

old male butterfly *Bicyclus anynana* has a more reproductive success than young males [7]. These results suggest that male investment in reproductive traits increase with aging in pre-copulatory sexual selection. Therefore, investment in post-copulatory sexual selection by males may also increase with age, similarly to pre-copulatory sexual selection.

On the other hand, male investment in pre- and post-copulatory sexual selection does not always coincide. For example, male investment in reproduction has often been reported to be a trade-off between pre- and post-copulation in many animal species [11-14]. Therefore, if investment in pre-copulatory sexual selection increases with male age, investment in post-copulatory sexual selection may decrease with male age.

The quality of gametes, however, declines with age and male senescence has been suggested to have profound evolutionary and ecological consequences on gamete performance and fitness [15]. In particular, new mutations have been shown to accumulate in the germline with age [16, 17], thereby decreasing the genetic quality of gametes [15, 18]. Selection experiments in the fruit fly (*Drosophila melanogaster*) have revealed that some aspects of sperm competitive ability decline with age [19, 20]. Reduced sperm competition success associated with male age has also been demonstrated in the bulb mite *Rhizoglyphus robini* and in the spider *Pholcus phalangioides* [21, 22]. In the hide beetle (*Dermestes maculatus*) found that old males were poor competitors against intermediate-age males, although they were slightly favoured towards young males [23]. However, sperm competition success in vitro did not correlate with male age in the sockeye

salmon (*Oncorhynchus nerka*) [24]. Moreover, in cricket *Teleogryllus oceanicus*, very young males had a greater proportion of nonviable sperm than middle-aged and older males [25]. Therefore, effects of male age on post-copulatory sex selection remains unclear and additional empirical studies are needed [21].

Another previous study suggested that the interaction between male mating history and male aging is important to post-copulatory sexual selection in male. For example, in the eastern mosquitofish (*Gambusia holbrooki*), among the same old males, males who experienced many copulations showed an increase in paternity success, while males who did not experience copulation showed a decrease in paternity success [26]. Thus, ~~focusing on~~ the interaction between aging and mating history in male may affect ~~to~~ post-copulatory sexual selection. However, few empirical studies have focused on the interaction between aging and mating history of male in post-copulatory sexual selection.

Here, we investigated the influence~~s~~ of male aging on male paternity success in the red flour beetle *Tribolium castaneum*. The *T. castaneum* infests stored products [27]. Many aspects of its reproduction are well understood, and it has advanced to become a major model organism in studies of post copulatory sexual selection [28, 29]. *T. castaneum* lives in high-density populations and both sexes copulate promiscuously and frequently [30-32]. During mating, males transfer sperm to the female, and females can store sperm for several months within an elaborate spermatheca [33, 34]. ~~That is,~~ a large number of previous studies have been conducted on post copulatory sexual selection, such as sperm competition and CFC. Therefore, it is a suitable study animal to demonstrate the relationship

between male aging and post copulatory sexual selection. To test the effect of interaction between aging and mating history of males on post-copulatory sexual selection, we also compared paternity success between males who experienced some copulations and males who did not experience copulation at old age. As in previous study, if older males who experienced copulations have an advantage in post-copulatory sexual selection, *T. castaneum* males who experienced copulations previously increase their paternity success with age than males who did not experience copulation.

Materials and Methods

Insects

The *T. castaneum* beetle culture used in this study has been maintained in the laboratory for more than 40 years, reared with a mixture of whole meal (Yoshikura Shokai, Tokyo) enriched with brewer's yeast (Asahi Beer, Tokyo), and maintained at 28°C with a 16 h photoperiod (lights on at 07:00, lights off at 23:00). Because this species is a stored grain pest, these conditions closely mirror the native environment of these beetles.

To compare the sperm competitiveness among males of various ages, we measured the paternity. Although broods may be sired by multiple males, the last male to mate fathers the majority of the offspring in the subsequent clutch [35]. Therefore, this study focused on sperm offence (P2 —fertilization success when the focal male is the second of two males to mate with a female). The focal males

and females used in this experiment belonged to a wild-type strain with brown body coloration. Competitor (non-focal) males were homozygous mutants for an autosomal, semidominant black body color allele, which is a phenotype that frequently used as a marker in sperm competition studies in *T. castaneum* [28]. We used focal male1, which was subjected to experiments from 10 days of age and allowed to mate every 100 days thereafter, and focal male2 which was aged without mating until it reached 300 days old. We used 10-day-old brown females. They were virgin and reared under monosexual condition until used for the experiment. Because we were evaluating the competitive ability of male beetle sperm in the male population at various ages, we used male black and brown beetles at various ages. We used 10-day-old beetles as the youngest individuals. This was considering the sexual maturation periods of *T. castaneum* [28].

Paternity success

We randomly collected virgin mutant black males and virgin brown females and placed them into a circular plastic container (10 mm in diameter) in pairs with food. The pair was allowed to mate freely for 24 h. After 24 h, the black male was removed from the container, and one focal male was put into the container and paired with a female that had already mated with a black strain male. Similarly, the pair was allowed to mate freely for 24 h. Because *T. castaneum* males often fail to mate, the pairs were placed together ~~for a period of time~~ and allowed to copulate freely without controlling the number of copulations. Thus, we mated two males separately with one female, but did not measure the exact number of copulations~~. Incidentally, several studies have investigated paternity in the same manner as this study~~ [36, 37]. Therefore, we considered that there are no major

problems with this methodology. After mating was completed, females were isolated in a plastic container (45 mm in diameter, 10 mm in height) with sufficient food and allowed to lay eggs for 10 days. Progeny was maintained at 28°C for 30 days to allow them to develop into adults, and the adult body color was scored to assign paternity and generate P2 scores. Males that had measurable paternity success at 10 days old were subjected to repeated paternity success measurement at 100, 200, and 300 days old. Due to the reproductive lifespan of *T. castaneum* being 0.5-1 year [38], measurement of paternal success in this study were conducted up to 300 days of age. When neither of the competing males sired offspring (i.e., female fertility was zero), the data were removed from the analysis as we could not be certain if the copulations were successful and resulted in sperm transfer or storage as females may also influence sperm retention. Because about one quarter males had died before day 300, the number of males who measured paternity success at 300 days old was fewer than that at 10- day-old males. During the experiment period, focal male 1 was kept separated in a sell plate with a sufficient amount of feed for survival to prevent contacting other individuals. All experiments were performed between 12:00 to 18:00 in a room maintained at 28°C.

To investigate of effects of interaction between aging and mating history on their paternity success in male, we also measured paternity success at 300 days old in males who did not experience copulation until 300 days old. In this experiment, paternity success was measured in the same method described above.

Statistical analysis

To analysis of paternity success, we used a generalized linear mixed model with binomial distribution because our data are binary data with either brown or black beetles. In this analysis, male age (10, 100, 200, and 300 days old) is an explanatory variable, and male ID is a random effect.

Paternity success at 300 days of age was compared between males whose paternity had been measured multiple times (mated male) and males whose paternity success had never been measured before (naive male); comparisons with the control males were conducted for each of the 10- and 300-day-old males, respectively. In this study, approximately 46% of all males died before 300 days. Thus, paternity success may be influenced through differences in longevity among males. To test this effect, the paternity success was determined at the last measurement before the focal male died was collected as the “paternity success at older age” (i.e., if males die at 150 days old, the paternity at 100 days old was used and if males lived passed the 300 days, we use the paternity success at 300 days old). We compared paternity success at older age and paternity success at young (10 days old).

All analyses were conducted in R ver.4.1.0 [39], using the statistical packages *lme4* [40] and *car* [41].

Results

Figure 1 shows paternity success of the males at each measured age. There is a significant effect of age on paternity success; paternity success significantly

decreased with male age ($\chi^2_{3,296} = 338.91$, $p < 0.0001$; Fig. 1). Figure 2 shows paternity success in the younger and older males. Paternity success of older males was significantly lower than that of younger males ($\chi^2_{1,152} = 315.18$, $p < 0.0001$; Fig. 2). Figure 3 shows paternity success of young male, old mated male, and old naive male. There is a significant effect of treatment on paternity success ($\chi^2_{2,233} = 236.17$, $p < 0.0001$); paternity success of young male was significantly higher than both old males, and paternity success of old mated males was significantly lower than old naive males (Fig. 3).

Discussion

We tested whether aging in *T. castaneum* males affected post-copulatory sexual selection. ~~As shown in Figure 1,~~ our results exhibited that paternity success decreased with male aging during the 300 days experimental period. This result suggests that males are at ~~a~~ disadvantage in post-copulatory sexual selection as they age. On the other hand, when comparing paternity between aged males who had copulated more than once and those who had not copulated at all, the paternity rate was higher in aged males who had copulated less than in aged males who had copulated more. The results suggest that older males with less mating experience invest more resources in reproduction and that the interaction between male aging and mating experience greatly influences male post-copulatory sexual selection. Although a previous study has conducted similar experiments [26], our study is the first to find increased paternity success in old males who did not experience copulation.

Effects of male aging to paternity success

A theoretical study suggested that males can increase their fitness by increasing their investment in reproductive traits with age [9], and empirical studies supporting this have been reported in several species [7, 8, 10, 42]. However, in this study, male paternity success of *T. castaneum* decreased with their male age. ~~In the results shown in Figure 1~~, individuals that died in the middle of the 300 day experimental period only had their paternity up to the time of death reflected in the results. Therefore, the effect of male longevity cannot be excluded from the results because only the paternity results of long-lived males are included as the age progresses. Therefore, paternity just before the death of the male (paternity at 300 days of age for males that lived until 300 days) was compared with paternity of young males, but paternity was still higher in young males (Fig. 2). Similar phenomena have also been reported in other animal species [19-23]. One cause of the age-related decline in male paternity may sperm quality. Previous study has reported that male sperm quality declines with males age [15]. Therefore, the sperm quality of males in *T. castaneum* may also have decreased with their age, which may have caused male paternity to decrease with male age. In this study, we also measured paternity of males that had not experienced mating for 300 days to eliminate the effect of repeated paternity measurements. Indeed, it is suggested that sperm quality declines with male age, as paternity was significantly lower in 300 days old males than in young males (Fig. 3). On the other hand, the paternity of naïve males was significantly higher than that of males with repeated paternity measurements (Fig. 3), suggesting that the effect on paternity was greater with number of copulations than with male aging.

Other possible explanation for our finding is that male investment in post-

copulatory sexual selection may have decreased as males aged. In many animals, males require investment in pre-copulatory sexual selection (e.g., investing in male–male combat for mating opportunities with females and ornament traits or courtship behaviours for female mating choice). Conversely, males that increased number of copulations often decreased their paternity, which has been suggested to be caused by trade-offs in resource allocation between pre- and post-copulatory sexual selection [43-45]. Since previous studies have suggested that investment in pre-copulatory sexual traits increase with aging [8, 10, 42], investment in post-copulatory sexual traits may decrease with male aging via the resource allocation trade-off. However, *T. castaneum* males do not have exaggerated weapon traits, do not exhibit violent male–male combat, and they have no flamboyant ornament traits. A previous study reported that males with high mobility had increased number of copulations than males with low mobility in *T. castaneum* [46]. Furthermore, another study revealed that males with low mobility had increased their paternity compared to males with high mobility of this beetle species [47]. Thus, in *T. castaneum* males, it is assumed that high mobility and searching for females, instead of male-male combat is important to increase the number of copulations, and that paternity decreased as a result of investment in mobility. Therefore, *T. castaneum* males may also have increased investment in pre-copulatory sexual selection with their age, resulting in increased mobility and decreased paternity with male age.

Furthermore, our findings may had been influenced by cryptic female choice. Cryptic female choice (sperm choice) is a post-copulatory sexual selection in which females control sperm use after copulation. This function is suggested in *T.*

castaneum [48]. Because offspring from old males may have low viability due to accumulation of mutations in the germ cells [49], females may refuse fertilization with old male sperm and precede it with young male sperm. That is, the result of this study may not be caused by the actual investment changes in males but may rather be the caused by cryptic female choice. As discussed below, male age alone is unlikely to be an important indicator of female choice, since paternity was significantly higher among males who never mated than among males whose paternity was repeatedly measured, even among males of the same age of 300 days.

There is also the possibility that the reduction in male paternity with age may be due to a decline in male performance. Previous studies have reported that behavioural performance declines with age in insect species [50-52]. This suggests that age-related damage to the morphological traits such as legs and wings has an effect on male paternity [53]. During copulation in *T. castanum*, males often exhibit a rubbing behaviour in which they mount the female from behind and rub the female's body with their legs [54]. Previous studies have been suggested that rubbing behavior by males may affect their paternity [28, 55]. It is considered that damage to legs with age in males may have had a negative effect on these copulatory behaviours and result in a decrease in their paternity success.

Interaction between aging and mating experiment in male

A recent study has reported that older males with more copulation experience exhibited higher paternity than older males with less copulation experience in *G.*

holbrooki [26]. This suggests that the interaction with male mating history also has an effect on their paternity not only male aging. However, although our study also revealed the significant effect of the interaction between aging and mating history ~~in male~~ on their paternity in *T. castaneum*, older males who experienced some copulations exhibited lower paternity than older males who did not experience copulation until experiment. Thus, our result differed from this previous study. Males of *G. holbrooki* used this previous study exhibited male-male combat fiercely for mating opportunities [56]. That is, males who experienced a lot of copulations may good male for females. On the other hand, in *G. cornutus*, it has been reported that females do not always prefer large weapon males as the dominant in male-male combat [57]. Conversely, *T. castaneum* male does not exhibited male-male combat fiercely for females, thus they are a highly promiscuous species [28]. This suggests that the effects of interaction between mating experience and aging in males on their paternity may differ between species. ~~That is,~~ male mating history may not be an indicator for male quality. In any case, it is suggested that male aging has a greater effect on their paternity than the abundance of male mating experience in *T. castaneum*. Males that never copulate until old age are expected to invest more resources in the few remaining reproductive opportunities than males that have copulated many times in the past. This may be the one reason why males who did not have the opportunity to mate until 300 days of age were higher paternity success than those who had mated before ~~in our results~~.

Our study revealed the effects of aging on paternity in males of *T. castaneum*, a model organism for post-copulatory sexual selection. However, there remain

many unclear factors that cannot be verified in this study. For example, it is necessary to compare the actual sperm quality between young and old males. It is also important to examine the relationship between locomotion activity and aging, which is largely related to paternity in this species, the relationship between cryptic female choice and male aging, and age-related leg damage in males. Although this study focused on P2 values because sperm attack is more prevalent in this species, it is also necessary to focus on sperm defense (that is, P1 values). It is important to conduct these investigations in the future to understand post copulatory sexual selection.

Acknowledgements

Dr. Yukio Yasui made valuable comments on this study. This work was supported by JSPS KAKENHI Grant Numbers 22K20664 and 23K14264 to K.M.

Reference

1. Andersson M. Sexual selection. Sexual Selection: Princeton University Press; 1994.

2. Darwin C. The descent of man, and selection in relation to sex. London, John Murry; 1871.

3. Thornhill R. Cryptic Female Choice and Its Implications in the Scorpionfly Harpobittacus nigriceps. The American Naturalist. 1983;122(6):765-88. doi: 10.1086/284170.

4. Eberhard W. Female Control: Sexual Selection by Cryptic Female Choice: Princeton University Press; 1996.

5. Firman RC, Gasparini C, Manier MK, Pizzari T. Postmating Female Control: 20 Years of Cryptic Female Choice. Trends Ecol Evol. 2017;32(5):368-82. doi: https://doi.org/10.1016/j.tree.2017.02.010.

6. Enquist M, Leimar O. The evolution of fatal fighting. Anim Behav. 1990;39(1):1-9. doi: https://doi.org/10.1016/S0003-3472(05)80721-3.

7. Fischer K, Perlick J, Galetz T. Residual reproductive value and male mating success: older males do better. Proceedings of the Royal Society B: Biological Sciences. 2008;275(1642):1517-24. doi: doi:10.1098/rspb.2007.1455.

8. Kemp DJ. Sexual selection constrained by life history in a butterfly. Proceedings of the Royal Society of London Series B: Biological Sciences. 2002;269(1498):1341-5. doi: doi:10.1098/rspb.2002.2000.

9. Kokko H. Evolutionarily stable strategies of age-dependent sexual advertisement. Behav Ecol Sociobiol. 1997;41(2):99-107.

10. Okada K, Katsuki M, Kiyose K, Okada Y. Older males are more competitive in male fights and more aggressive toward females in the broad-horned flour beetle *Gnatocerus cornutus*. Behav Ecol Sociobiol. 2020;74(3):1-10.

11. Danielsson I. Antagonistic pre–and post–copulatory sexual selection on male body size in a water strider (Gerris lacustris). Proceedings of the Royal Society of London Series B: Biological Sciences. 2001;268(1462):77-81.

12. Fu P, Neff BD, Gross MR. Tactic–specific success in sperm competition. Proceedings of the Royal Society of London Series B: Biological Sciences. 2001;268(1472):1105-12.

13. Preston BT, Stevenson IR, Pemberton JM, Wilson K. Dominant rams lose out by sperm depletion. Nature. 2001;409(6821):681-2.

14. Warner RR, Shapiro DY, Marcanato A, Petersen CW. Sexual conflict: males with

highest mating success convey the lowest fertilization benefits to females. Proceedings of the Royal Society of London Series B: Biological Sciences. 1995;262(1364):135-9. doi: doi:10.1098/rspb.1995.0187.

15. Pizzari T, Dean R, Pacey A, Moore H, Bonsall MB. The evolutionary ecology of pre- and post-meiotic sperm senescence. Trends Ecol Evol. 2008;23(3):131-40. doi: https://doi.org/10.1016/j.tree.2007.12.003.

16. Glaser RL, Jabs EW. Dear Old Dad. Science of Aging Knowledge Environment. 2004;2004(3):re1-re. doi: doi:10.1126/sageke.2004.3.re1.

17. Risch N, Reich EW, Wishnick MM, McCarthy JG. Spontaneous mutation and parental age in humans. Am J Hum Genet. 1987;41(2):218-48. PubMed PMID: 3618593; PubMed Central PMCID: PMCPMC1684215.

18. Hansen TF, Price DK. Good genes and old age: Do old mates provide superior genes? J Evol Biol. 1995;8(6):759-78. doi: https://doi.org/10.1046/j.1420-9101.1995.8060759.x.

19. Service PM, Fales AJ. Evolution of delayed reproductive senescence in male fruit flies: sperm competition. In: Rose MR, Finch CE, editors. Genetics and Evolution of Aging. Dordrecht: Springer Netherlands; 1994. p. 130-44.

20. Uglem I, Galloway T, Rosenqvist G, Folstad I. Male dimorphism, sperm traits and immunology in the corkwing wrasse (*Symphodus melops* L.). Behav Ecol Sociobiol. 2001;50(6):511-8. doi: 10.1007/s002650100392.

21. Radwan J. Male age, germline mutations and the benefits of polyandry. Ecol Lett. 2003;6(7):581-6. doi: https://doi.org/10.1046/j.1461-0248.2003.00484.x.

22. Schäfer MA, Uhl G. Determinants of paternity success in the spider *Pholcus phalangioides* (Pholcidae: Araneae): the role of male and female mating behaviour. Behav Ecol Sociobiol. 2002;51(4):368-77. doi: 10.1007/s00265-001-0448-9.

23. Jones TM, Featherston R, Paris DBBP, Elgar MA. Age-related sperm transfer and sperm competitive ability in the male hide beetle. Behav Ecol. 2007;18(1):251-8. doi: 10.1093/beheco/arl077.

24. Hoysak DJ, Liley NR, Taylor EB. Raffles, roles, and the outcome of sperm competition in sockeye salmon. Can J Zool. 2004;82(7):1017-26. doi: 10.1139/z04-073.

25. García-González F, Simmons LW. Sperm Viability Matters in Insect Sperm Competition. Curr Biol. 2005;15(3):271-5. doi: https://doi.org/10.1016/j.cub.2005.01.032.

26. Aich U, Bonnet T, Head ML, Jennions MD. Disentangling the effects of male age

and mating history: Contrasting effects of mating history on precopulatory mating behavior and paternity success. Evolution. 2021;75(11):2867-80. doi: 10.1111/evo.14371.

27. Beeman RW. Distribution of the Medea factor M4 in populations of Tribolium castaneum (Herbst) in the United States. J Stored Prod Res. 2003;39(1):45-51. doi: https://doi.org/10.1016/S0022-474X(02)00016-4.

28. Fedina TY, Lewis SM. An integrative view of sexual selection in *Tribolium* flour beetles. Biol Rev Camb Philos Soc. 2008;83(2):151-71. doi: 10.1111/j.1469-185X.2008.00037.x. PubMed PMID: 18429767.

29. Pai A, Bernasconi G. Polyandry and female control: the red flour beetle Tribolium castaneum as a case study. Journal of Experimental Zoology Part B: Molecular and Developmental Evolution. 2008;310B(2):148-59. doi: https://doi.org/10.1002/jez.b.21164.

30. Pai A, Yan G. Rapid female multiple mating in red flour beetles (Tribolium castaneum). Can J Zool. 2003;81(5):888-96. doi: 10.1139/z03-070.

31. Lewis SM. Multiple mating and repeated copulations: effects on male reproductive success in red flour beetles. Anim Behav. 2004;67(4):799-804. doi: https://doi.org/10.1016/j.anbehav.2003.05.013.

32. Grazer VM, Martin OY. Elevated temperature changes female costs and benefits of reproduction. Evol Ecol. 2012;26(3):625-37. doi: 10.1007/s10682-011-9508-4.

33. Qazi MCB, Herbeck JT, Lewis SM. Mechanisms of Sperm Transfer and Storage in the Red Flour Beetle (Coleoptera: Tenebrionidae). Ann Entomol Soc Am. 1996;89(6):892-7. doi: 10.1093/aesa/89.6.892.

34. Bernasconi G, Brostaux Y, Meyer EP, Arnaud L. Do spermathecal morphology and inter-mating interval influence paternity in the polyandrous beetle Tribolium castaneum? Behaviour. 2006;143(5):643-58. doi: https://doi.org/10.1163/156853906776759538.

35. Lewis SM, Jutkiewicz E. Sperm precedence and sperm storage in multiply mated red flour beetles. Behav Ecol Sociobiol. 1998;43(6):365-9. doi: 10.1007/s002650050503.

36. Bernasconi G, Keller L. Female polyandry affects their sons' reproductive success in the red flour beetle *Tribolium castaneum*. J Evol Biol. 2001;14(1):186-93.

37. Michalczyk Ł, Millard AL, Martin OY, Lumley AJ, Emerson BC, Gage MJG. Experimental evolution exposes female and male responses to sexual selection and conflict in *Tribolium castaneum*. Evolution. 2011;65(3):713-24. doi: 10.1111/j.1558-5646.2010.01174.x.

38. Sokoloff A. The biology of *Tribolium*, with special emphasis on genetic aspects: Oxford University Press; 1974.

39. Team RC. R: A language and environment for statistical computing. R Foundation for Statistical Computing, Vienna, Austria. URL https://www.R-project.org/. 2021.

40. Bates D, Maechler M, Bolker B, Walker S. Fitting Linear Mixed-Effects Models Using lme4. J Stat Software. 2015.

41. Fox J, Weisberg S. An [R] companion to applied regression. Third edition. Thousand Oaks CA: Sage. 2019.

42. Rohwer S, Fretwell SD, Niles DM. Delayed Maturation in Passerine Plumages and the Deceptive Acquisition of Resources. The American Naturalist. 1980;115(3):400-37. doi: 10.1086/283569.

43. Eberhard W. Female control: sexual selection by cryptic female choice: Princeton University Press; 1996.

44. Parker GA. Sperm competition and its evolutionary consequences in the insects. Biological reviews. 1970;45(4):525-67.

45. Simmons LW. The evolution of polyandry: an examination of the genetic incompatibility and good-sperm hypotheses. J Evol Biol. 2001;14(4):585-94. doi: https://doi.org/10.1046/j.1420-9101.2001.00309.x.

46. Matsumura K, Miyatake T. Differences in Attack Avoidance and Mating Success between Strains Artificially Selected for Dispersal Distance in *Tribolium castaneum*. PLoS ONE. 2015;10(5):e0127042. Epub 20150513. doi: 10.1371/journal.pone.0127042. PubMed PMID: 25970585; PubMed Central PMCID: PMCPMC4430303.

47. Matsumura K, Archer CR, Hosken DJ, Miyatake T. Artificial selection on walking distance suggests a mobility-sperm competitiveness trade-off. Behav Ecol. 2019;30(6):1522-9. doi: 10.1093/beheco/arz110.

48. Fedina TY, Lewis SM. Proximal traits and mechanisms for biasing paternity in the red flour beetle *Tribolium castaneum* (Coleoptera: Tenebrionidae). Behav Ecol Sociobiol. 2006;60(6):844-53. doi: 10.1007/s00265-006-0228-7.

49. Johnson SL, Gemmell NJ. Are old males still good males and can females tell the difference? Bioessays. 2012;34(7):609-19. doi: https://doi.org/10.1002/bies.201100157.

50. Gargano JW, Martin I, Bhandari P, Grotewiel MS. Rapid iterative negative geotaxis (RING): a new method for assessing age-related locomotor decline in *Drosophila*. Exp Gerontol. 2005;40(5):386-95. doi: https://doi.org/10.1016/j.exger.2005.02.005.

51. Leffelaar D, Grigliatti T. Age-dependent behavior loss in adult *Drosophila melanogaster*. Dev Genet. 1983;4(3):211-27. doi: https://doi.org/10.1002/dvg.1020040307.

52. Martinez VG, Javadi CS, Ngo E, Ngo L, Lagow RD, Zhang B. Age-related changes in climbing behavior and neural circuit physiology in *Drosophila*. Developmental Neurobiology. 2007;67(6):778-91. doi: https://doi.org/10.1002/dneu.20388.

53. Ridgel AL, Ritzmann RE. Insights into age-related locomotor declines from studies of insects. Ageing Research Reviews. 2005;4(1):23-39. doi: https://doi.org/10.1016/j.arr.2004.08.002.

54. Wojcik DP. Mating Behavior of 8 Stored-Product Beetles (Coleoptera: Dermestidae, Tenebrionidae, Cucujidae, and Curculionidae). The Florida Entomologist. 1969;52(3):171-97. doi: 10.2307/3493853.

55. Edvardsson M, Göran A. Copulatory courtship and cryptic female choice in red flour beetles <i>Tribolium castaneum</i>. Proceedings of the Royal Society of London Series B: Biological Sciences. 2000;267(1443):559-63. doi: doi:10.1098/rspb.2000.1037.

56. McPeek MA. Mechanisms of sexual selection operating on body size in the mosquitofish (*Gambusia holbrooki*). Behav Ecol. 1992;3(1):1-12. doi: 10.1093/beheco/3.1.1.

57. Okada K, Katsuki M, Sharma MD, House CM, Hosken DJ. Sexual conflict over mating in *Gnatocerus cornutus*? Females prefer lovers not fighters. Proceedings of the Royal Society B: Biological Sciences. 2014;281(1785):20140281. doi: doi:10.1098/rspb.2014.0281.

Figures and Tables

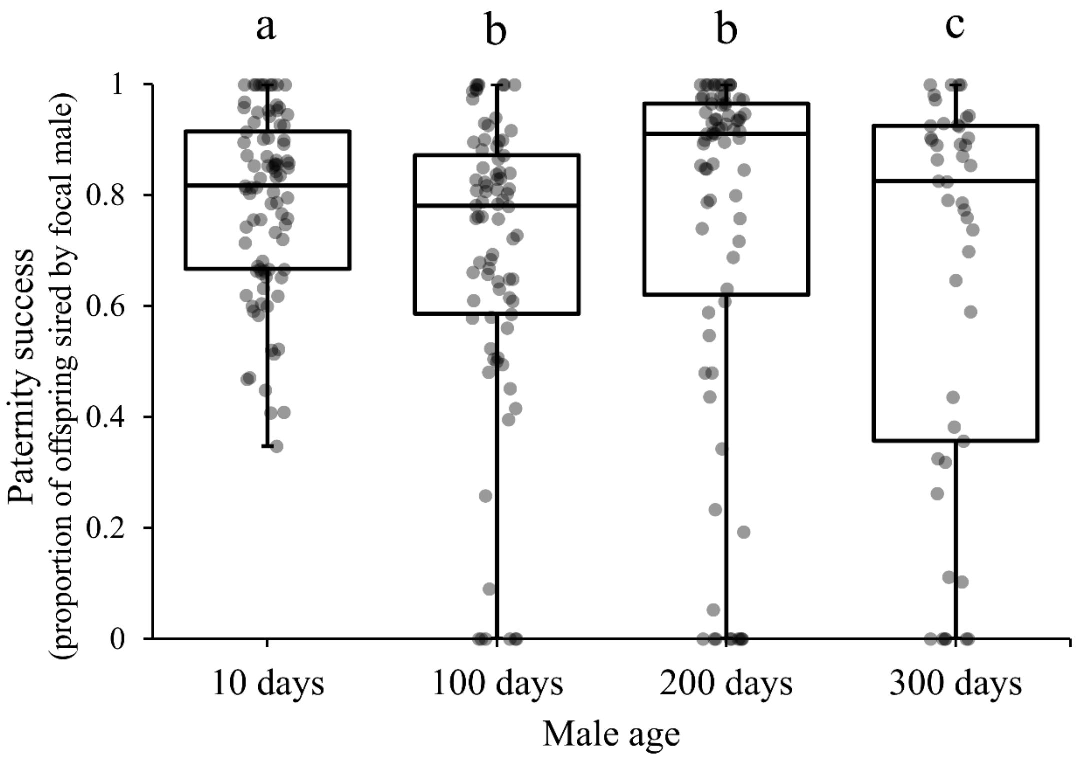

Figure 1. Box plots of paternity success (proportion of offspring sired by the male) of *T. castaneum* males at 10, 100, 200, and 300 days old, respectively. Box plots show median and interquartile range of the data. There are significantly differences among different characters (Bonferroni correction).

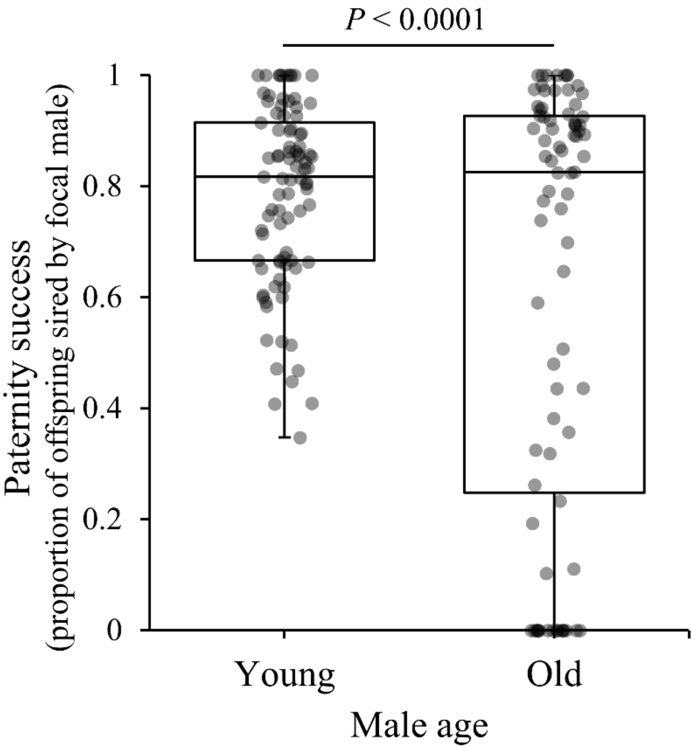

Figure 2. Box plots of paternity success (proportion of offspring sired by the male) of *T. castaneum* males young (10 days old) and old (prior to death at any age of days). Box plots show median and interquartile range of the data.

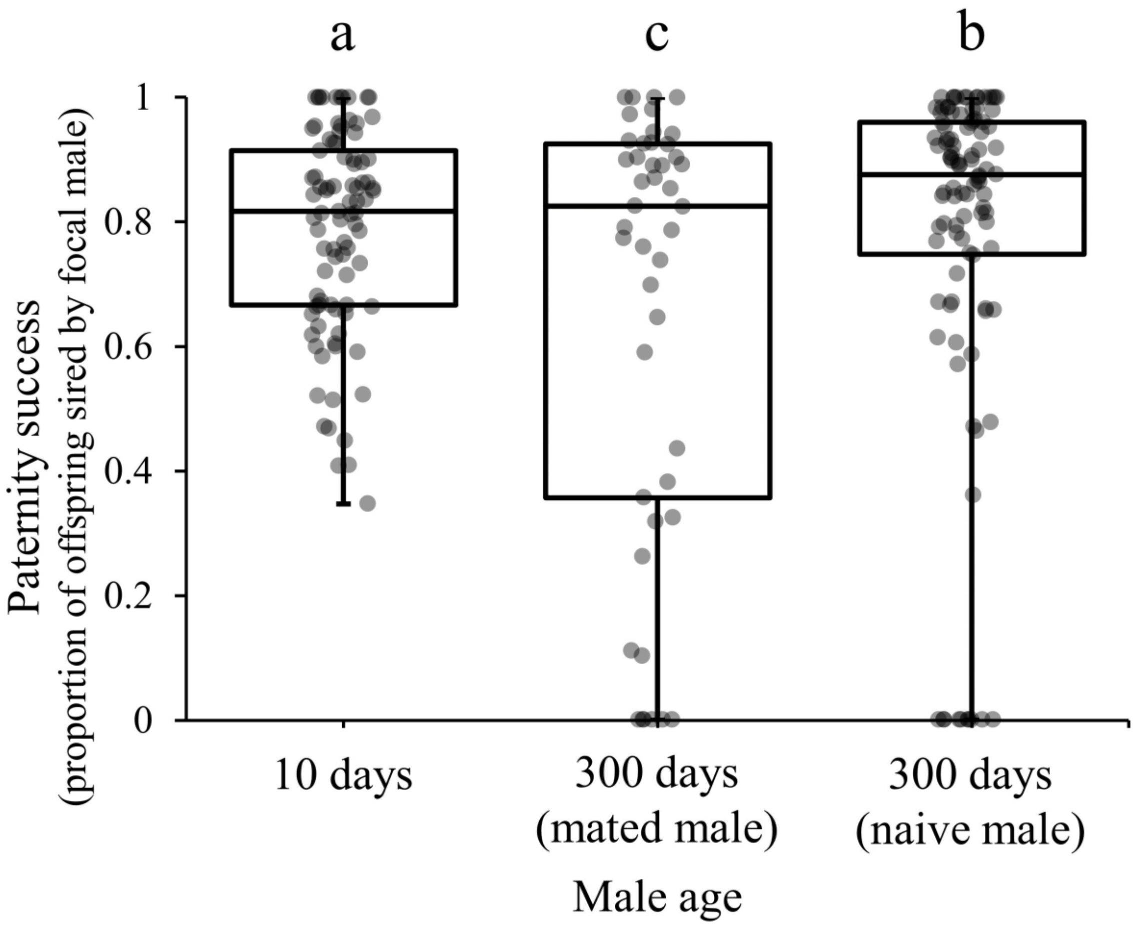

Figure 3. Box plots of paternity success of young male (10 days old), old male who experienced some copulations, and old male who did not experience copulation until measurement. Box plots show median and interquartile range of the data. There are significantly differences among different characters (Bonferroni correction).

Click here to access/download;Figure;Figure 1.tif

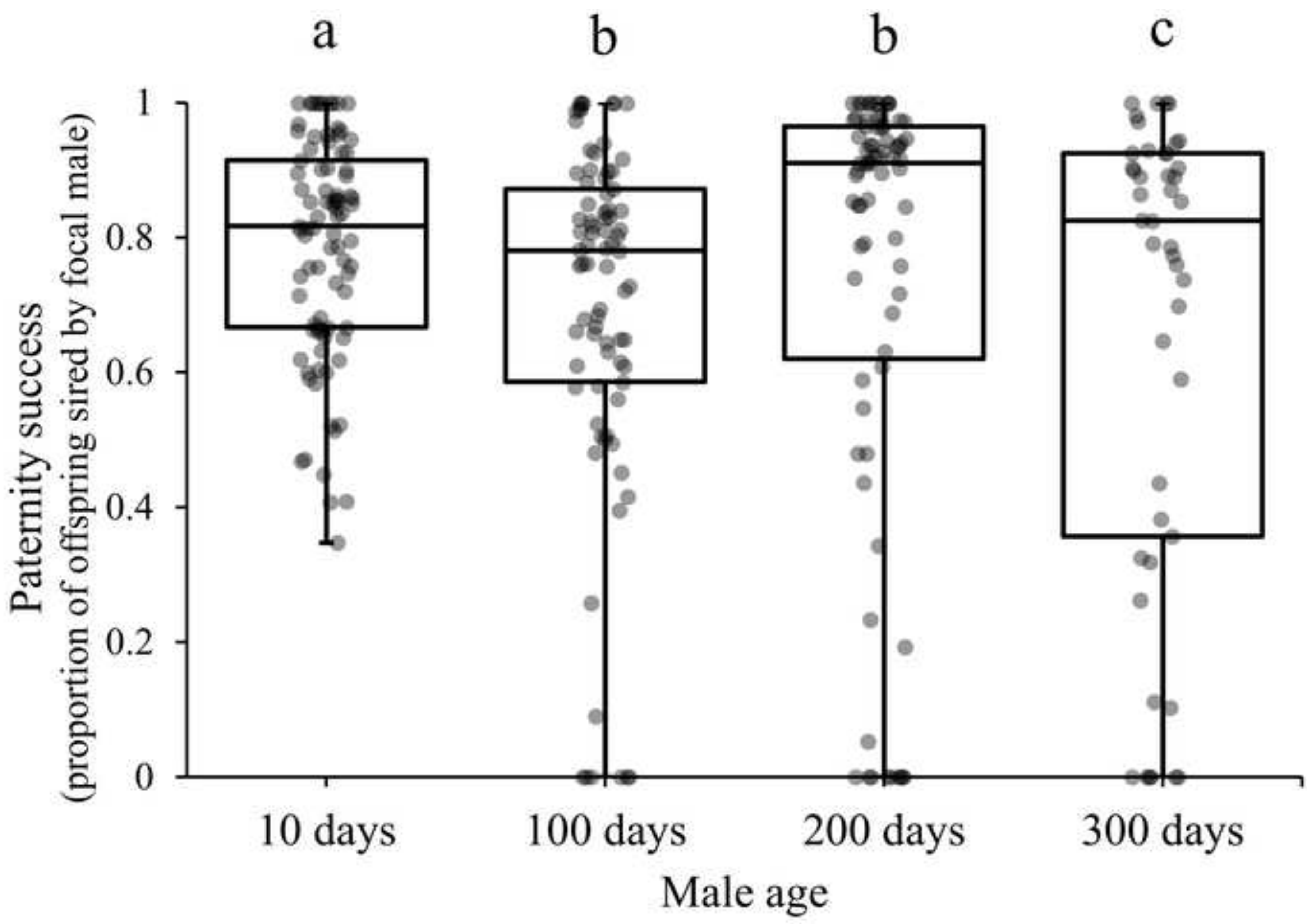

Click here to access/download;Figure;Figure 2.tif

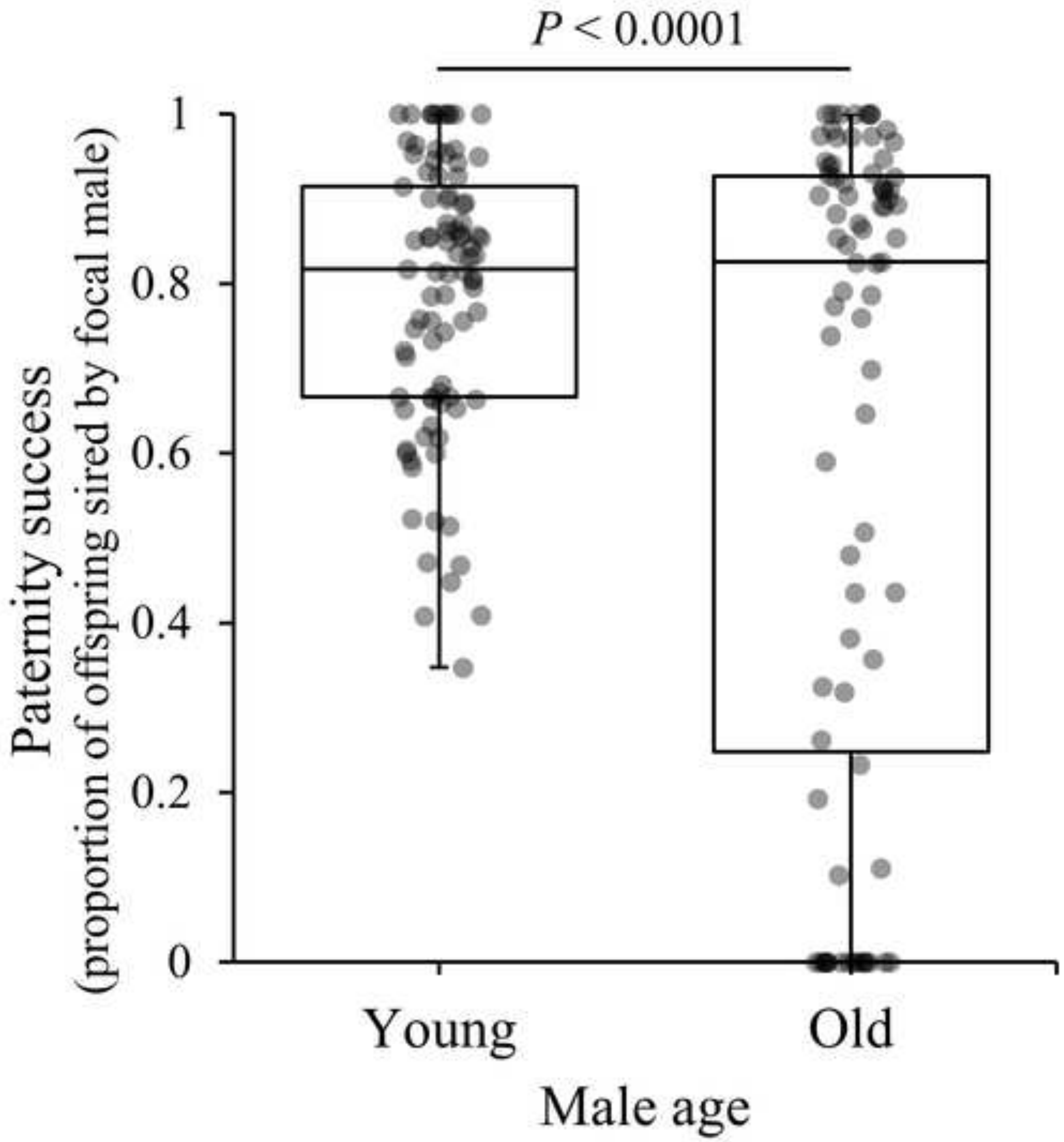

Click here to access/download;Figure;Figure 3.tif

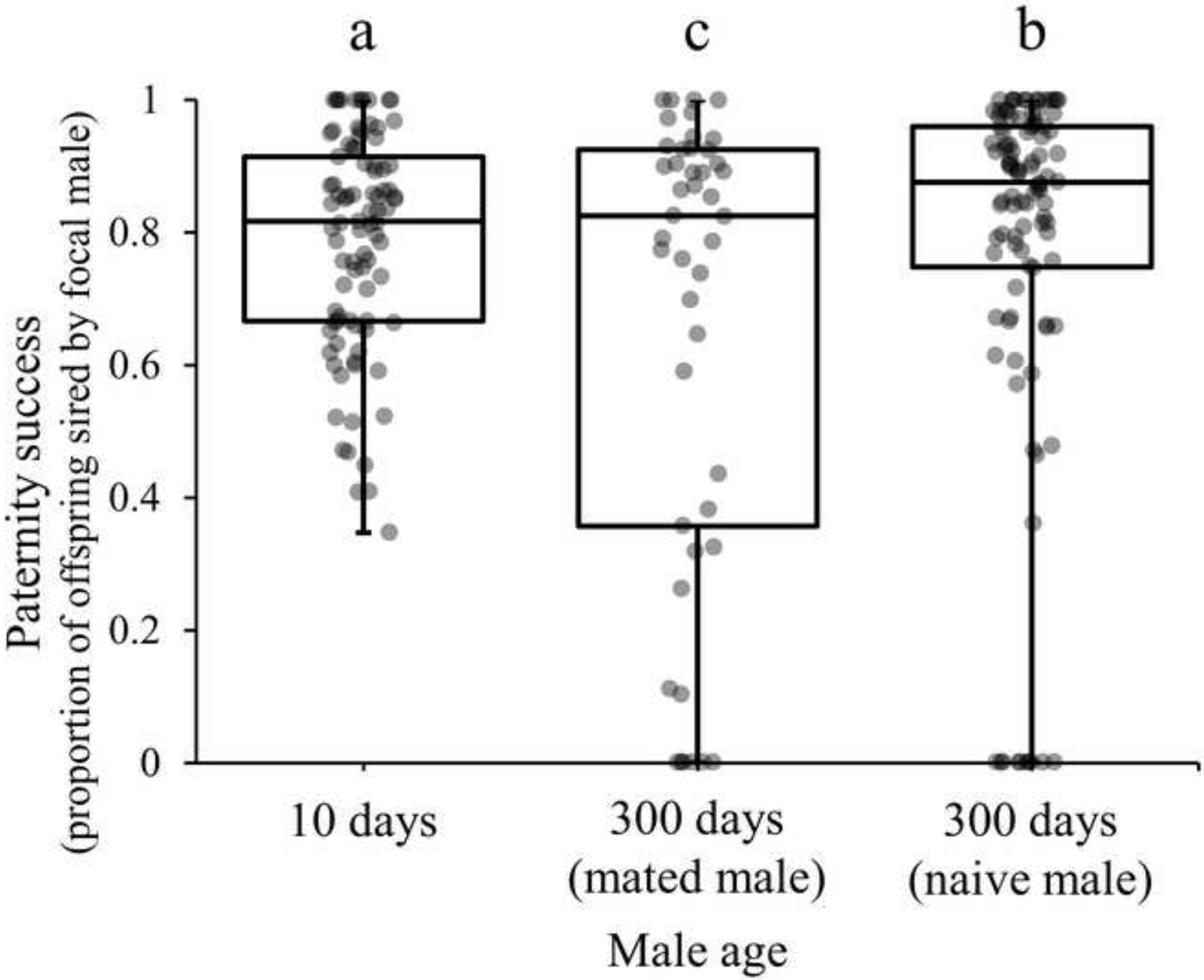

Supplement: Attachment — Submitted filename: PONE-D-24-05330_reviewer-2.pdf [file pone.0316008.s006.pdf]
