# Supplementary material for: Influences of aging and mating history in males on paternity success in the red flour beetle Tribolium castaneum
Source: PLoS One. 2024 Dec 23;19(12):e0316008. doi: 10.1371/journal.pone.0316008 (PMC11665990; doi:10.1371/journal.pone.0316008)
Supplement: Attachment — Submitted filename: Review POLS ONE 11.03.2024 comments to authors.docx [file pone.0316008.s005.docx]

*Comments to the Author*

In their study titled ‘Influences of aging and mating history in males on their paternity in the red flour beetle *Tribolium castaneum*’ the authors test if there is an interaction between the effects of male aging and mating history on paternity success in the model insect species *T. castaneum*. They found that paternity success significantly decreased with age, but surprisingly old males that did not mate before had significantly higher paternity success compared to experienced old males.

Overall, I think this study uses a simple, but effective and elegant study design to address a very relevant question in sexual selection and reproductive biology. What is the effect of male ageing and sperm aging on paternity? Nevertheless, I believe that the manuscript could be substantially improved in clarity.

Unfortunately, the authors state: ‘data available on request from the author’. Stating this is against journals policy (for good reason, I think) and I believe that the manuscript should only be published if the authors have also published the underlying data (or given very strong reasons why this is not possible). ‘Data available on request from the author’ has been shown to be harmful praxis that often leads to inaccessible data (also see Tedersoo et al 2021).

Major comments

Abstract: I made some wording suggestions (see below) and I think that the results are currently hard to follow when only reading the abstract.

Introduction: Overall, the structure is good, but I think that the hypotheses could be more clearly defined and the expectations more thoroughly explained. I also believe that the prominent focus on male-male combat is a bit misplaced, as the data do not address this topic. I would argue, that instead it could be more clearly defined what effects sperm aging and aging in general might have on the different treatments.

Methods: The methods need some substantial clarifications for me. Please see my comments below. In particularly sample sizes are missing in the whole manuscript. Only giving dfs for tests is not sufficient in my opinion. Please give starting sample sizes and individual, final sample sizes for each treatment, preferably (also) added to the figures. I think it is also crucial to more clearly state how the competitors (i.e. black) individuals were aged and maintained. Finally, I have reservations concerning the egg laying period and particularly the censoring of unsuccessful focals in the first mating test (see detailed comments below).

Results: Though this is nicely concise, I think the results could be described in slightly more detail. While I like the presented figures it is hard to appreciate the data in the current presentation. I think giving means and/or percentages in reduction or increase in the results text would greatly help with that. In addition, a table with full statistics for the individual post-hoc comparisons presented in the figures, could be worthwhile (preferentially including effect sizes and/or means + error).

Discussion: The discussion nicely summarizes the main findings and is structured well. Nevertheless, I think that the hypotheses explaining the results are not all straightforward and fully discussed. Please see my detailed comments for thoughts and suggestions on alternative interpretations.

Minor comments

Short title: ‘Influences of aging and mating history on their paternity’ – not clear what ‘their’ refers to here. Actually, I think that the ‘their’ could be removed here and also in the main title.

L20: Change to ‘males’?

L24: Remove ‘to’?

L30ff: This seems contradictory to me: Did mating history affect paternity or not? If ‘naive old males showed significantly higher paternity success than mated old males’, then mating history did matter, right? Please clarify. I think one can only clearly understand what is meant here when reading the whole manuscript, but the abstract should be understandable without that.

L34: Change to ‘mating history affected paternity success’?

L40f: For me, natural selection is ultimately also about increasing reproductive success. Maybe have a look at Shuker & Kvarnemo (2021) for some suggestions how to define sexual selection (though I do not fully agree with their definition either …).

L46: Please add a citation for ‘premating sexual selection’.

L46: No only in polyandrous species not all sperm are used for fertilization. The vast majority of sperm do not fertilize any eggs. I think ‘not the sperm of every male is used …’ might be meant here.

L49: Long and confusing sentence. Please revise.

L53: The acronym ‘CFC’ is never explained. Also, it is only used twice in the manuscript. I would avoid the use of acronyms where possible.

L59: ‘One factor affecting male investment in reproductive traits is male aging. ‘ Please add a reference for this.

L60: Change to: ‘... die in male-male combat ...’?

L65ff: I think this might be true for potentially terminal investments, like in the example above, but certainly not for all male reproductive traits (like sperm production). Offspring produced in early life should be just as valuable as offspring produced in late life. I would even argue the opposite is often true: Aging and deaths will make reproduction late in life less likely. Hence, investing early in life has larger mean pay-off.

L88ff: I think the recent meta-analysis on sperm aging by Sanghvi et al. (2024) would be very relevant here. (I am not one of the authors.)

L111: Remove ‘to’?

L128ff: Long and confusing sentence. Please revise.

L129: Which ‘previous study’? Please cite.

L149: What kind of ‘wild-type strain’? What is its origin?

L153: Ages are in relation to eclosion date? So, 10 days means 10 days post eclosion? Please specify.

L155: ‘brown’ meaning wild-type, correct? Maybe call them ‘wild-type’ thought to clarify?

L158: Could you please specify this? Were all beetles from the same generation (hence age-synchronised at the beginning of the experiment)?

L168ff: Not sure why this is a reason not to control for the number of matings. Behavioural data would surely still be informative. Still, I think it is fair to say that there likely was at least one successful copulation in each pair.

L173f: Omit? Just because something has been done, it does not mean it is not problematic. I think is fine, but this sentence is not needed in my opinion.

L176: Why only 10 days? Do you think that letting them lay eggs for longer might change things? Is there any evidence that P2 changes over time?

L178f: I am not sure that excluding males that did not reproduce at day 10 from the dataset does not bias the data. At later points, males with zero offspring were still included, right? If success is compared over time this might bias data towards a higher mean offspring number at day 10 compared to later time points, I think.

L188: What about before the first trial? Were males separated early on? I ask because males have frequent same-sex copulations which might affect the results. For the same reason: Please specify how the competitors were kept. Where they also separated until trials? Also, where they always the same age or were as old as the focal? Was this the same competitor for each male or always an new one?

L188: Apologies, but I do not know what a ‘sell plate’ is. Could you please specify?

L189: Change to ‘… contact with …’.

L195: Related to comment above: Were these males kept individually until day 300?

L200: Were the model residuals normally distributed and not over-dispersed? Otherwise ‘quasi-binomial’ might give a more reliable fit.

L222: This sentence is more a legend then a results sentence. Maybe rather start with a summary of the most important result (like the next sentence)?

L222: Change to ‘… was a significant effect ….’. Please check whole section is written in past tense.

Line 224f/227f: Again, I think this sentence is not needed. Saying what the figure shows is the object of a figure legend.

L228: Please clarify what is meant by ‘treatment’ here.

L230f: Could you please illustrate this in more detail? How large was the difference between naïve old males and experienced old males? I would also be interested in the statistics on this, as this is very interesting and, to me, surprising.

Figures: I like the figures! But please add sample sizes here or elsewhere. Also, Bonferroni correction is mentioned here, but not in the Statistical methods, I believe.

L235: Omit: ‘As shown in Figure 1’? Personally, I would throughout avoid direct references to figures or tables in the discussion.

L244: Maybe mention that this study was done in a different species?

L249ff: As in the introduction, I find it surprising that the authors focus so much on this paper. It is a great paper, but in my reading deals foremost with advertisement-, not with sperm competition, traits. Hence, I do not think that the presented data are a good fit to test these models. Indeed, the data does more closely fit what I would predict under sperm aging effects (see e.g. Sanghvi et al. 2924).

L255ff: I am no sure about this analysis, I have to say. What matters in the end is each individual’s lifetime reproductive success. Maybe it would be more informative to look at the total reproductive success of all individuals and correlate that with individual survival (10, 100, 200 or 300 days)? This could show if lifetime reproductive success trades-off with lifespan.

L260: Again, see Sanghvi et al. 2024.

L268ff: And on the other hand …? Seems something is missing here.

L270f: ‘… suggesting that the effect on paternity was greater with number of copulations than with male aging.’ I personally do not think this is likely. 4 days of mating interactions over 300 days seems very low for a species like *T. castaneum*…

L273ff: This section is again on the general decline of P2 with age, right? Maybe clarify this, as I thought first this would be a continuation of the discussion on why naïve males did better then experienced males.

L291: I find these trade-off arguments not very convincing, given that pre-copulatory traits (particularly mobility) should also increase the number of - or chances for mating. This should also increase P2 and repeated mating should be beneficial in sperm competition as well as sperm quality or quantity per mating. I think the authors argument only strictly holds if the number of matings were equal for each trail, which was likely not the case. I think this could be discussed as a limitation.

L297f: Add references for cryptic female choice definition?

L301f: Here it would be crucial to know more about the competitor (i.e. black) male (see comments on methods). This only holds if competitors were all young males. Might one use different competitor ages in the future to test this hypothesis?

L310: Initially unclear what is meant by ‘performance’. Maybe substitute with ‘courtship behaviour’?

L335: Don’t think ‘thus’ is correct here.

L340ff: I am struggling to understand the mechanism for this. Are males that copulated before ‘exhausted’? Again, given the high mating rates in *T. castaneum* this seems unlikely to me. If this is really about differential investment, how are naïve males supposed to know when to invest more. In other words: How does a male know that he needs to invest most resources late in life compared to early? Is it not most likely for a male red flour beetle to mate hundreds or thousands of times before reaching the age of 300 days - so how did this plasticity in investment into post-copulatory traits evolve?

L344: Change to ‘… had higher paternity success …’?

L354: Change ‘sperm attack’ to ‘last-male sperm precedence’?

L356f: True, but maybe it is more interesting to finish with a sentence about what the present data can teach us? - They are interesting after all.

References

Sanghvi, K., Vega-Trejo, R., Nakagawa, S. et al. Meta-analysis shows no consistent evidence for senescence in ejaculate traits across animals. Nat Commun 15, 558 (2024). https://doi.org/10.1038/s41467-024-44768-4

Shuker DM, Kvarnemo C. The definition of sexual selection. Behav Ecol. 2021 Aug 7;32(5):781-794. doi: 10.1093/beheco/arab055.

Tedersoo, L., Küngas, R., Oras, E. et al. Data sharing practices and data availability upon request differ across scientific disciplines. Sci Data 8, 192 (2021). https://doi.org/10.1038/s41597-021-00981-0
