# Supplementary material for: Influences of aging and mating history in males on paternity success in the red flour beetle Tribolium castaneum
Source: PLoS One. 2024 Dec 23;19(12):e0316008. doi: 10.1371/journal.pone.0316008 (PMC11665990; doi:10.1371/journal.pone.0316008)
Supplement: Attachment — Submitted filename: Responses to Editors and Reviewers comments_20240828.docx [file pone.0316008.s007.docx]

Dear Dr. Basana Gowda G,

First of all, thank you very much for extending the deadline for submitting our revised manuscript. Thank you for giving us the opportunity to repost our manuscript (PONE-D-24-05330). We corrected the manuscript as much as possible to accommodate the helpful comments from the excellent reviewers. There is no doubt that our revised manuscript is better than before. We describe our response immediately after each reviewer's comment. Our corrections in the main text are shown in red. Please confirm.

5. Review Comments to the Author

Reviewer #1: In their study titled ‘Influences of aging and mating history in males on their paternity in the red flour beetle Tribolium castaneum’ the authors test if there is an interaction between the effects of male aging and mating history on paternity success in the model insect species T. castaneum. They found that paternity success significantly decreased with age, but surprisingly old males that did not mate before had significantly higher paternity success compared to experienced old males.

Response: Thank you for many useful comments to our manuscript. We corrected the manuscript as much as possible to accommodate the helpful comments from your comments. There is no doubt that our revised manuscript is better than before. We describe our response immediately after each your comment. Our corrections in the main text are shown in red. Please confirm.

Overall, I think this study uses a simple, but effective and elegant study design to address a very relevant question in sexual selection and reproductive biology. What is the effect of male ageing and sperm aging on paternity? Nevertheless, I believe that the manuscript could be substantially improved in clarity.

Response: Thank you for your appreciation of our research. The reasons for this finding are summarized in the end of Discussion (Lines: 421-435).

Unfortunately, the authors state: ‘data available on request from the author’. Stating this is against journals policy (for good reason, I think) and I believe that the manuscript should only be published if the authors have also published the underlying data (or given very strong reasons why this is not possible). ‘Data available on request from the author’ has been shown to be harmful praxis that often leads to inaccessible data (also see Tedersoo et al 2021).

Response: Thank you for your important comments. All data used in this paper will be made available to anyone as supplementary material.

Major comments

Abstract: I made some wording suggestions (see below) and I think that the results are currently hard to follow when only reading the abstract.

Response: Thank you for your useful comments to this Abstract. We corrected these sentences following your suggestions (Lines: 20-37).

Introduction: Overall, the structure is good, but I think that the hypotheses could be more clearly defined and the expectations more thoroughly explained. I also believe that the prominent focus on male-male combat is a bit misplaced, as the data do not address this topic. I would argue, that instead it could be more clearly defined what effects sperm aging and aging in general might have on the different treatments.

Response: We corrected some sentences related to male-male combat in Introduction following your suggestions (Lines: 42-146).

Methods: The methods need some substantial clarifications for me. Please see my comments below. In particularly sample sizes are missing in the whole manuscript. Only giving dfs for tests is not sufficient in my opinion. Please give starting sample sizes and individual, final sample sizes for each treatment, preferably (also) added to the figures. I think it is also crucial to more clearly state how the competitors (i.e. black) individuals were aged and maintained. Finally, I have reservations concerning the egg laying period and particularly the censoring of unsuccessful focals in the first mating test (see detailed comments below).

Response: We corrected some sentences in Materials and Methods (Lines: 148-262). We added sample size (Lines: 218-219, 236).

Results: Though this is nicely concise, I think the results could be described in slightly more detail. While I like the presented figures it is hard to appreciate the data in the current presentation. I think giving means and/or percentages in reduction or increase in the results text would greatly help with that. In addition, a table with full statistics for the individual post-hoc comparisons presented in the figures, could be worthwhile (preferentially including effect sizes and/or means+error).

Response: We corrected sentences in Results section, and we added mean and se of each paternity success (Lines: 264-294).

Discussion: The discussion nicely summarizes the main findings and is structured well. Nevertheless, I think that the hypotheses explaining the results are not all straightforward and fully discussed. Please see my detailed comments for thoughts and suggestions on alternative interpretations.

Response: We corrected some sentences in Discussion following your comments (Lines: 297-435).

Minor comments

Short title: ‘Influences of aging and mating history on their paternity’ – not clear what ‘their’ refers to here. Actually, I think that the ‘their’ could be removed here and also in the main title.

Response: Removed “their”.

L20: Change to ‘males’?

Response: Corrected.

L24: Remove ‘to’?

Response: Removed “to”.

L30ff: This seems contradictory to me: Did mating history affect paternity or not? If ‘naive old males showed significantly higher paternity success than mated old males’, then mating history did matter, right? Please clarify. I think one can only clearly understand what is meant here when reading the whole manuscript, but the abstract should be understandable without that.

Response: Corrected.

L34: Change to ‘mating history affected paternity success’?

Response: Corrected.

L40f: For me, natural selection is ultimately also about increasing reproductive success. Maybe have a look at Shuker & Kvarnemo (2021) for some suggestions how to define sexual selection (though I do not fully agree with their definition either …).

Response: Corrected (Lines: 42-45).

L46: Please add a citation for ‘premating sexual selection’.

Response: Corrected (Line: 50).

L46: No only in polyandrous species not all sperm are used for fertilization. The vast majority of sperm do not fertilize any eggs. I think ‘not the sperm of every male is used …’ might be meant here.

Response: Corrected following your suggestions (Lines: 51).

L49: Long and confusing sentence. Please revise.

Response: Corrected (Lines: 55-60).

L53: The acronym ‘CFC’ is never explained. Also, it is only used twice in the manuscript. I would avoid the use of acronyms where possible.

Response: Corrected (Line: 57).

L59: ‘One factor affecting male investment in reproductive traits is male aging. ‘ Please add a reference for this.

Response: Added references (Line: 66).

L60: Change to: ‘... die in male-male combat ...’?

Response: Corrected following your suggestion (Line: 67).

L65ff: I think this might be true for potentially terminal investments, like in the example above, but certainly not for all male reproductive traits (like sperm production). Offspring produced in early life should be just as valuable as offspring produced in late life. I would even argue the opposite is often true: Aging and deaths will make reproduction late in life less likely. Hence, investing early in life has larger mean pay-off.

Response: As you say, the length of a male's life span may greatly affect paternity. So, we looked at the relationship between male longevity and paternity, the relationship between male body size and paternity, and the relationship between male body size and longevity. Added those results (Lines: 290-294) and discussion (Lines: 361-372).

L88ff: I think the recent meta-analysis on sperm aging by Sanghvi et al. (2024) would be very relevant here. (I am not one of the authors.)

Response: We added sentences (Lines: 104-105, 320-322).

L111: Remove ‘to’?

Response: Corrected.

L128ff: Long and confusing sentence. Please revise.

Response: Corrected.

L129: Which ‘previous study’? Please cite.

Response: Corrected these sentences (Lines: 139-146).

L149: What kind of ‘wild-type strain’? What is its origin?

Response: Corrected these sentences (Lines: 158-169).

L153: Ages are in relation to eclosion date? So, 10 days means 10 days post eclosion? Please specify.

Response: Corrected (Line: 185).

L155: ‘brown’ meaning wild-type, correct? Maybe call them ‘wild-type’ thought to clarify?

Response: We changed from brown to wild-type.

L158: Could you please specify this? Were all beetles from the same generation (hence age-synchronised at the beginning of the experiment)?

Response: Corrected (Lines: 158-169, L172-236).

L168ff: Not sure why this is a reason not to control for the number of matings. Behavioural data would surely still be informative. Still, I think it is fair to say that there likely was at least one successful copulation in each pair.

Response: We added sentence (Lines: 172-187).

L173f: Omit? Just because something has been done, it does not mean it is not problematic. I think is fine, but this sentence is not needed in my opinion.

Response: We removed.

L176: Why only 10 days? Do you think that letting them lay eggs for longer might change things? Is there any evidence that P2 changes over time?

Response: In this experiment, about 80 eggs were laid over 10 days. Therefore, it is considered that the number of eggs is not low. It would be interesting to know whether the P2 value changes over time, but in this experiment the spawning period was set to 10 days because of labor difficulties (Lines: 203-206).

L178f: I am not sure that excluding males that did not reproduce at day 10 from the dataset does not bias the data. At later points, males with zero offspring were still included, right? If success is compared over time this might bias data towards a higher mean offspring number at day 10 compared to later time points, I think.

Response: Males with zero offspring at the time of paternity measurement at 10 days of age are likely to be sterile. Conversely, males that do not have zero offspring when paternity is measured at 10 days of age are at least not sterile. Therefore, we did not use males with zero offspring at the time of paternity measurement at 10 days of age for subsequent experiments, but used males with non-zero offspring for subsequent experiments.

L188: What about before the first trial? Were males separated early on? I ask because males have frequent same-sex copulations which might affect the results. For the same reason: Please specify how the competitors were kept. Where they also separated until trials? Also, where they always the same age or were as old as the focal? Was this the same competitor for each male or always an new one?

Response: In the experiment, the males were isolated so as not to contact each other (Lines: 222-223).

L188: Apologies, but I do not know what a ‘sell plate’ is. Could you please specify?

Response: Corrected (Line: 221).

L189: Change to ‘… contact with …’.

Response: Corrected (Line: 223)

L195: Related to comment above: Were these males kept individually until day 300?

These males also were also kept individually (Lines: 230-236).

L200: Were the model residuals normally distributed and not over-dispersed? Otherwise ‘quasi-binomial’ might give a more reliable fit.

Response: As you say, the DP was calculated and it turned out to be overdispersion, since both models showed values of about 20. Therefore, we used the estimated dispersion parameter to correct the variance (Collett, 2002; Faraway, 2016) (Lines: 255-259, Fig. 1-3, Table 1).

L222: This sentence is more a legend then a results sentence. Maybe rather start with a summary of the most important result (like the next sentence)?

Response: We corrected sentences in Results section (Lines: 264-294)

L222: Change to ‘… was a significant effect ….’. Please check whole section is written in past tense.

Response: We corrected these sentences (Lines: 264-294).

Line 224f/227f: Again, I think this sentence is not needed. Saying what the figure shows is the object of a figure legend.

Response: We corrected these sentences (Lines: 264-294).

L228: Please clarify what is meant by ‘treatment’ here.

Response: We corrected these sentences (Lines: 264-294).

L230f: Could you please illustrate this in more detail? How large was the difference between naïve old males and experienced old males? I would also be interested in the statistics on this, as this is very interesting and, to me, surprising.

Response: We corrected these sentences (Lines: 264-294).

Figures: I like the figures! But please add sample sizes here or elsewhere. Also, Bonferroni correction is mentioned here, but not in the Statistical methods, I believe.

Response: We added sample sizes (Fig 2-4, 218-219), and sentences for multiple analysis (Lines: 257-259).

L235: Omit: ‘As shown in Figure 1’? Personally, I would throughout avoid direct references to figures or tables in the discussion.

Response: Corrected these sentences (Lines: 298-313).

L244: Maybe mention that this study was done in a different species?

Response: Corrected (Lines: 298-313).

L249ff: As in the introduction, I find it surprising that the authors focus so much on this paper. It is a great paper, but in my reading deals foremost with advertisement-, not with sperm competition, traits. Hence, I do not think that the presented data are a good fit to test these models. Indeed, the data does more closely fit what I would predict under sperm aging effects (see e.g. Sanghvi et al. 2924).

Response: We corrected Discussion (Lines: 298-435).

L255ff: I am no sure about this analysis, I have to say. What matters in the end is each individual’s lifetime reproductive success. Maybe it would be more informative to look at the total reproductive success of all individuals and correlate that with individual survival (10, 100, 200 or 300 days)? This could show if lifetime reproductive success trades-off with lifespan.

Response: We tested this additionally because, as you say, male longevity and paternity may be related. Added results (Lines: 290-294) and discussion (Lines: 361-372).

L260: Again, see Sanghvi et al. 2024.

Response: Added (Line: 322).

L268ff: And on the other hand …? Seems something is missing here.

Response: We corrected these sentences (Lines: 298-435).

L270f: ‘… suggesting that the effect on paternity was greater with number of copulations than with male aging.’ I personally do not think this is likely. 4 days of mating interactions over 300 days seems very low for a species like T. castaneum…

Response: Thank you for your important comments. It may be very important for a male to have even one successful mating. Indeed, there should be a large difference between males that have had only four mating opportunities but have already produced some offspring and males that have yet to produce any offspring.

L273ff: This section is again on the general decline of P2 with age, right? Maybe clarify this, as I thought first this would be a continuation of the discussion on why naïve males did better then experienced males.

Response: We corrected Discussion (Lines: 298-435).

L291: I find these trade-off arguments not very convincing, given that pre-copulatory traits (particularly mobility) should also increase the number of - or chances for mating. This should also increase P2 and repeated mating should be beneficial in sperm competition as well as sperm quality or quantity per mating. I think the authors argument only strictly holds if the number of matings were equal for each trail, which was likely not the case. I think this could be discussed as a limitation.

Response: We corrected Discussion (Lines: 298-435).

L297f: Add references for cryptic female choice definition?

Response: We have added definitions to the introduction

L301f: Here it would be crucial to know more about the competitor (i.e. black) male (see comments on methods). This only holds if competitors were all young males. Might one use different competitor ages in the future to test this hypothesis?

Response: We used black male with random age (Line: 191).

L310: Initially unclear what is meant by ‘performance’. Maybe substitute with ‘courtship behaviour’?

Response: We corrected Discussion (Lines: 298-435).

L335: Don’t think ‘thus’ is correct here.

Response: Corrected.

L340ff: I am struggling to understand the mechanism for this. Are males that copulated before ‘exhausted’? Again, given the high mating rates in T. castaneum this seems unlikely to me. If this is really about differential investment, how are naïve males supposed to know when to invest more. In other words: How does a male know that he needs to invest most resources late in life compared to early? Is it not most likely for a male red flour beetle to mate hundreds or thousands of times before reaching the age of 300 days - so how did this plasticity in investment into post-copulatory traits evolve?

Response: To repeat my previous comment, we believe that the experience of copulation is more important than the frequency of copulation. However, as you say, this worm has the potential to experience hundreds of copulations before it reaches 300 days of age, so it would be interesting to see the effect of increasing the number of copulations.

L344: Change to ‘… had higher paternity success …’?

Response: Corrected.

L354: Change ‘sperm attack’ to ‘last-male sperm precedence’?

Response: We corrected Discussion (Lines: 298-435).

L356f: True, but maybe it is more interesting to finish with a sentence about what the present data can teach us? - They are interesting after all.

Response: Corrected (Lines: 421-435).

References

Sanghvi, K., Vega-Trejo, R., Nakagawa, S. et al. Meta-analysis shows no consistent evidence for senescence in ejaculate traits across animals. Nat Commun 15, 558 (2024). https://doi.org/10.1038/s41467-024-44768-4

Shuker DM, Kvarnemo C. The definition of sexual selection. Behav Ecol. 2021 Aug 7;32(5):781-794. doi: 10.1093/beheco/arab055.

Tedersoo, L., Küngas, R., Oras, E. et al. Data sharing practices and data availability upon request differ across scientific disciplines. Sci Data 8, 192 (2021). <https://doi.org/10.1038/s41597-021-00981-0>

Reviewer #2: The subject of the article is original and shall be published, however, there are some flaws and room to make it better and more easily understandable to the scientific readers.

Response: Thank you for many useful comments to our manuscript. We corrected the manuscript as much as possible to accommodate the helpful comments from your comments. There is no doubt that our revised manuscript is better than before. We describe our response immediately after each your comment. Our corrections in the main text are shown in red. Please confirm.

There is a need to some English polishing in the article.

Response: We have revised the manuscript following your and other reviewers' comments. If it is judged that there is still a problem with the text of this manuscript, we will ask an English reviewer to proofread it.

The abstract is clear and concise.

Response: Thank you.

In the introduction there are some references needed that are missing, regarding some sentences.

Response: We added some references in Introduction.

When mentioning a species for the first time, the descriptors' name shall be added.

Response: Added descriptor’s name (Line: 29).

There is some concern on whether results from studies with different animals (for example, fishes, birds, humans) may apply to discuss the results obtained here. The suggestion would be to add the information on what animal the reference refers to, at least.

Response: We have added a sentence indicating which biological group the cited references pertain to.

I suggest separating the objectives in two, and rewrite them in the end of the Introduction (1-influence of age; 2-influence of previous copulations, or as authors find better).

Response: Thank you for your comment. We corrected these sentences following you and other reviewer’s comments (Lines:42-146).

The methodology needs some rearrangement to be more clear, and delete repetitions. Please describe how you will measure paternity success as soon as you refer to this. It is not clear to the reader if the "proportion of offspring sired by the male" refers to number of broods or to number of insects in each brood, please clarify.

Response: Corrected (Lines: 148-262).

Lines 147 and 148 (with P2) are very confusing, please re-write this to be more clear. A scheme would be a nice addition, stating the beetles used (focal male1, male 2, females, and their age, and, non focal males).

Response: We added figure indicating experimental design of this study (Fig. 1).

The fact that these beetles are from laboratory rearing for 40 years may interfere with their behaviour? Would like that authors could discuss this a bit.

Response: We added sentences following your suggestion (Lines: 417-419).

Also, could the mutant beetles have some type of limitation or advantage in terms of sexual behaviour compared to the focal ones? Was this previously tested by the authors or in the literature?

Response: Your comment is certainly important. However, there have been many studies in which the black strain of the worm has been used in the same way (Line: 165). Therefore, we have decided that you can ignore the issues you are concerned about.

I suggest to arrange methodology and results within the 2 objectives set: 1) age vs success and 2) naive vs mated males (+ longevity/age).

Response: We added sentence (Lines: 148-262, 264-294).

A scheme or infographics would facilitate the understanding of the trials and its link to the objectives.

Response: We added figure indicating experimental design of this study (Fig. 1).

Regarding the statistical analysis, it misses the presentation of a more detailed numerical output of the model(s) done.

Data presentation with box and whisker plots is quite visual, but at least in the last one the differences are not clear, so I suggest putting a table instead, adding the statistical output similarly to the figure.

Response: We added more information of statistical analysis in Table 1.

Could Figure 1 and 2 be combined? Just a suggestion.

Response: Thank you for your suggestion. However, we think that it is better to separate Fig. 1 and Fig. 2 for the explanation, so we have not corrected it.

The described part of sperm competitiveness does not seem to be in the results (between focal and non focal males), or it is not clearly explained.

Response: We corrected some sentences in Materials & Methods (Lines: 171-236).

In the end of the discussion there is a reference to P2 (already described) and to P1, but this would need a little more attention, for example, in the introduction or methods, as it is not clear here.

Response: We added it in method section (Lines: 172-187).

Some further suggestions are done in the attached document.

Response: Thank you for your detailed comments in the PDF file. Below is a list of your comments and our response is written right after each comment.

L22: relatively to

Response: Corrected (Line: 22).

L26: different ages

Response: Corrected (Line: 28).

L30: in this study

Response: Corrected (Line: 32).

L69-71: not clear, please rewrite

Response: Corrected (Lines: 75-78).

L109: please define this here according to 26

Response: we defined (Lines: 117-119)

.

L115: In this study, the objective was to...

Response: Corrected (Line: 126).

L115: please define this. number of copulations? broods? descendants?

Response: we defined. (Lines: 127-128).

L117: ...is...

Response: Corrected (Line: 130).

L124: an adequate model

Response: Corrected (Line: 137).

L128-132: write this as objectives of the study

Response: Corrected (Lines: 140-147).

L141: mimic

Response: Corrected (Line: 156).

L147: please clarify this, it seems not complete

Response: Corrected (Lines: 181-182).

L163: clarify their age here

Response: Corrected (Lines: 192-193).

L176: an average number of progeny for brood would be a nice addition to the results

Response we added (Line: 208).

L178: focal or non focal? this clarification is needed through this part of the text

Response: Corrected (Line: 210).

L240: or no copulation

L242: or post copulatory strategies?

Response: Corrected these sentences.

L244: are the results of this study with an insect comparable with results of a study with a fish?

Response: Corrected (Lines: 314-318).

L245-246: maybe this finding should be stated in the abstract

Response: We added this to the abstract. (Lines: 37-38).

L253: without reaching

L255-256: mortality factor

L257: paternity success

L261: be

L266: ? please clarify

L266-271: please define paternity here (success, when previously defined before in the article. this needs re-phrasing

L273: what finding? please define

L285: showed higher

L311: sexual performance

L313: castaneum

Response: We corrected the entire text in response to these points.

L325: exerts

Response: Corrected (Line: 383).

L325: paternity, and not only male age.

L326: male aging

Response: We corrected the entire text in response to these points.

L330: in this

Response: Corrected (Line: 388).

L332: may be

Response: Corrected (Line: 390).

L337: success

Response: Corrected (Line: 396).

L338: for all species

Response: You commented 'for all species,' but we have revised it to 'in *T. castaneum*' How does that sound? (Line: 398).

L339: the results of this study suggest that

Response: We corrected the entire text in response to these points.

L343: a explanation for the fact that...

Response: Corrected (Line: 404).

L349: could not

Response: Corrected (Line: 417).

L352: paternity success

Response: We corrected the entire text in response to these points.

Regardless of all the observations I maintain that this is a very interesting subject that shall be published, however it needs major revisions.

Response: Thank you for your many useful comments.

Reviewer #3: The manuscript by Kawakami and Matsumura examines the effects of age on sperm competitive ability (offensive) of the red flour beetle Tribolium castaneum. The authors compared paternity success of young males (10 days-old) and old males (100, 200 and 300 days-old) by allowing the focal male to mate with a female for 24h after the female was first given 24h to mate with a competitor male. The authors also examined sperm competitive ability of naïve, old males (300 days-old).

The results suggest that old Tribolium castaneum males became less able to compete with young male sperm as they age. Several studies have demonstrated that old males are less able than their younger counterparts in several reproductive processes, including sperm competition, making the results reported here in agreement with other similar studies. However, I have some concerns about these results which I have outlined below.

Response: Thank you for many useful comments to our manuscript. We corrected the manuscript as much as possible to accommodate the helpful comments from your comments. There is no doubt that our revised manuscript is better than before. We describe our response immediately after each your comment. Our corrections in the main text are shown in red. Please confirm.

1. We are given very little information about mating behaviors in this species, particularly in information that may help the reader better interpret the reported results. Is spermatophore quality affected by male age as ejaculate quality is in Drosophila (https://doi.org/10.1016/j.exger.2017.12.013)? Further, can females influence the process of sperm storage in this species? Each of these may affect sperm quantity that enter the spermatheca which can alter sperm competition outcomes.

Response: It is unknown whether the quality of spermatophores in male *Tribolium castaneum* is affected by the age of the males. It is considered that females can regulate the amount of male spermatophores transferred to the spermatheca (Fedina & Lewis, 2006), which could potentially influence the outcome of sperm competition. The potential influence of females is discussed in the discussion section (Lines: 344-359).

2. How often do females remate? A quick glance at the literature suggests that females can mate multiple times within the first few hours after introduction of the male. Similarly, does male age affect their ability to court and inseminate females? Male mating frequency may decline with age. What is the mating frequency of young males compared to old males? If younger males’ mate at higher frequencies, then they may transfer larger sperm quantities, potentially making them better to sire more offspring. This could give the appearance of better sperm competitive ability, but this might be an indirect effect of the transfer of larger sperm quantities rather than a function of sperm quality.

Response: When the statistical analysis was redone with comments from other reviewers, there was no paternity difference between naive old and young males. The results suggest that sperm quality may not be affected by aging. I rewrote the text of the discussion (Lines: 298-435).

Also, if males mate less frequently than young males (due to declines in copulation attempts/success with age), a larger proportion of males wold have P2 scores of 0 with increasing male age. The increasing P2 scores of 0 seen in figure 1 may be indicative of worse sperm performance, or increased failure to mate. In the hide beetle Dermestes maculatus, males are less likely to transfer sperm as they age (https://doi.org/10.1093/beheco/arl077). This needs to be addressed.

Response: Again, the same old males that had never mated did not show reduced paternity. Therefore, this result is interesting because it suggests that aging does not reduce fertility. We have rewritten our considerations significantly (Lines: 298-435).

3. It is known if male age affects sperm quantity transferred? In the hide beetle Dermestes maculatus, old males that transfer sperm transfer similar quantities as young males (https://doi.org/10.1093/beheco/arl077). However, in other species such as Drosophila, males transfer less sperm with age (https://doi.org/10.1073/pnas.200905311). Interestingly, the age effect in Drosophila is only observed in males that had previous mating opportunities (similar to the experimental setup here), but is not seen in old, naïve males.

Response: We don't know the amount of ejaculation from this study, so it is our future task to investigate this (Lines: 408-415).

Thus, while it may be difficult to control for the number of copulations by a mating pair, it may be necessary potentially minimize indirect effects of male age that can complicate interpretation of reported results. Given that the focus is on sperm ability, aspects that can be influence paternity scores need to be controlled as much as possible.

Response: Thank you for your comment. These will be our future research subjects (Lines: 421-435).

4. It states in the ms that males that had paternity success at the initial mating were then mated every 100 days. Thus, it is possible to examine paternity success at each mating event? Do you see an overall decline with age? This may be indicative of a failure to mate with increased male age.

Response: Males with zero initial paternity were excluded from further experiments because they might be completely sterile.

5. The authors need to clearly state their sample sizes (preferably in the figures). Also, was this performed with only a single cohort of males?

Response: We specified the number of samples in the Figures and Methods section (Lines: 218-219, 236).

6. I do not understand Figure 2. The legend states that the “old” group comprises males “prior to death at any age of days”. Is this simply all the old males from Fig. 1 (that is, all males that lived to at least 100 days)? Or is it data from males that were mated at 100 or 200 days but did not live to the next mating age? This needs to be clarified.

Response: Figure 2 compares the paternity of focal male 1 at 10 days old with the paternity just before death. The age of focal male 1 when measuring paternity just before death varied among individuals. For example, focal male 1 that died at 150 days old had its paternity just before death measured at 100 days old. Similarly, focal male 1 that died at 250 days old had its paternity just before death measured at 200 days old (Lines: 585-592).

Reviewer #4: The manuscript titled "Influences of aging and mating history in males on their paternity in the red flour beetle Tribolium castaneum" aimed to evaluate. paternity success of males at each age in the red flour beetle Tribolium castaneum. To investigate effects of mating history, they also compared the paternity success between older males who experienced some copulations (mated male) and older males who did not experience copulation (naive male). Because the authors affirmed that "the relationship between male aging and paternity remains unclear.

Response: Thank you for many useful comments to our manuscript. We corrected the manuscript as much as possible to accommodate the helpful comments from your comments. There is no doubt that our revised manuscript is better than before. We describe our response immediately after each your comment. Our corrections in the main text are shown in red. Please confirm.

Studies related to the mating behavior of Tribolium castaneum have been extensively researched. Some articles in this area were found in my research and are not included in the discussion or Introduction.

Response: We referenced previous studies used *T. castaneum* (Lines: 153-154, 158-169, 172-181, 334-336).

I ask the authors what the added value of these studies is compared to previous ones? What significant contributions do they bring to the understanding of this species, for example, in population control, infestations, and mass rearing?

Response: We added sentences following your comments (Lines: 298-313, 421-435).

Before proceeding with a more detailed review, the authors should explain in the text what distinguishes this work from others.

A note: Tribolium castaneum is written in italics.

Response: Corrected.

Kentarou Matsumura

On behalf of all authors
