# Supplementary material for: Influences of aging and mating history in males on paternity success in the red flour beetle Tribolium castaneum
Source: PLoS One. 2024 Dec 23;19(12):e0316008. doi: 10.1371/journal.pone.0316008 (PMC11665990; doi:10.1371/journal.pone.0316008)
Supplement: Attachment — Submitted filename: Response to Reviewers_20241031.docx [file pone.0316008.s008.docx]

Dear Dr. Basana Gowda G,

Thank you for giving us the opportunity to repost our manuscript (PONE-D-24-05330R1). We corrected the manuscript as much as possible to accommodate the helpful comments from the excellent reviewers. There is no doubt that our revised manuscript is better than before. We describe our response immediately after each reviewer's comment. Reviewers pointed out that our English was poor, so we had an English proofreader correct the English in this manuscript. In this revised manuscript, our corrections are in red and corrections due to English proofreading are in blue. Please confirm.

Journal Requirements:

Please review your reference list to ensure that it is complete and correct. If you have cited papers that have been retracted, please include the rationale for doing so in the manuscript text, or remove these references and replace them with relevant current references. Any changes to the reference list should be mentioned in the rebuttal letter that accompanies your revised manuscript. If you need to cite a retracted article, indicate the article’s retracted status in the References list and also include a citation and full reference for the retraction notice.

Response: We could not figure out which “retracted papers” you pointed out. Could you please tell us which one is the “retracted paper”?

Additional Editor Comments:

Authors have not carefully attended to the comments raised by reviewers. Address them thoroughly.

Response: We did everything possible to address the points raised by the reviewers. Please take a moment to review the revised manuscript.

Reviewers' comments:

Reviewer #1: I would like to thank the Authors for their careful consideration of my comments and their replies.

I believe that the changes made improved the clarity of the manuscript, but I am afraid there are still instances, where there is work to be done.

Response: Thank you for many useful comments to our manuscript. We corrected the manuscript as much as possible to accommodate the helpful comments from your comments. There is no doubt that our revised manuscript is better than before. We describe our response immediately after each your comment. We had an English proofreader correct the English in this manuscript. In this revised manuscript, our corrections are in red and corrections due to English proofreading are in blue. Please confirm.

I think that the reasoning in the Introduction and Discussion is not always convincing and that the theory for sperm aging and male investment into pre-copulatory traits, post-copulatory traits and immunity/longevity need to be discussed in more detail if mentioned so prominently. I am surprised that the Authors discuss male strategies for investing into reproduction and longevity without once mentioning the word ‘trade-off’. I feel a better theoretical basis is needed to discuss the present findings.

Response: Thank you for your important remarks. You are right, the amount of investment in reproduction and survival in males is often expected to be a trade-off. However, what we looked at in this study was whether males that had the misfortune of never being able to mate until old age would increase their investment in reproduction. Therefore, we did not use the word “trade-off” in our discussion because it was difficult to explain the trade-off in terms of a simple investment in reproduction versus investment in longevity (Lines: 390-417).

I particularly appreciate, that the Authors decided to publish their data. I think this is valuable and good practice. To make this even better, I think a legend explaining the different variables in detail would be most useful. Additionally, I think that the body size measurements (presented in the Supplement) are missing in the data set. Please add these as well.

Response: We are sorry that we forgot to provide the data of body size and longevity. We added these data to the supplemental information.

Detailed comments

L26: Should be ‘… that investigated influences …’?

Response: Corrected (Line: 26).

L34: Should be ‘… suggest that an interaction…’?

Response: Corrected (Line: 35).

L42: Should be ‘Evolution is change in allele frequencies of a population over generations’? I don’t think ‘genetic frequency’ is the right term.

Response: Removed this phrase.

L53: Could be shortened to ‘This is called…’?

Response: That sentence was corrected by English reviewer (Line: 54).

L67-69: Please revise. ‘…are expected to obtain …’ and ‘… decrease investment into costly traits for reproduction …’?

Response: Corrected (Lines: 68-70).

L139-146: This is helpful, I think! Please make clearer that these are hypotheses (e.g. ‘their paternity success should increase with’ or ‘… is predicted to increase with…’

Response: Corrected (Lines: 143, 145-146).

L310: ’… used by fish …’ Should be ‘… in the fish …’?

Response: Corrected (Line: 319).

L348: ‘pre-copulatory sexual selection may increase with age’ – Selection was not measured here and I think it is not meant. I think it should be ‘Investment into pre-copulatory traits’.

Response: Corrected (Lines: 360-361).

L350: ‘pre-copulatory sexual selection may increase with age’ - Maybe the Authors could give an example which traits they have in mind here?

Response: Corrected these sentences (Lines: 362-365).

L379: Should be ‘influenced by the interaction’?

Response: Corrected (Line: 395).

L393-395: I am sorry, but I still don’t buy this argumentation. Survival and reproduction are interrelated and ultimately a combination of both makes up an individual’s fitness. Only survival without reproduction does not give any fitness returns. Likewise, only reproduction with little investment into survival is not a good strategy. A more nuanced discussion could help clarify this, I think.

The argumentation here boils down to a trade-off between reproductive traits and immunity. Still, the Authors fail to mention even the word ‘trade-off’ and do not seem to engage with the literature on this. E.g. What is the evidence that such a trade-off exists? For the present data it seems that there is no such trade-off (Fig S1). Did males in the treatment without matings until 300 days old survive better compared to the other treatment? I think that mating is just inherently costly to males in Tribolium (see Spatt 1980). One reason might be that they pass scarce resources (like water) to the female as a sort of nuptial gift with their ejaculate (see Moiron et al. 2022). Hence, the present results might not be a trade-off or a question of investment by the males, as the Authors seem to argue here, but a by-product of mating being costly.

Finally, I still think that the way the Authors argue, they make the assumption that males ‘know’ how many matings they can expect in the future and how long they might survive. But this information is not available to the individual, hence selection should favor an optimal strategy for a ‘mean Tribolium life-history’ and/or plastic adaptations depending on past experience.

Response: As you say, using the same word "fitness" for males in two different situations was likely to cause unnecessary confusion. Males that have already copulated need not rush to reproduce, so investment in risky reproduction may decrease with age. On the other hand, older males that have never mated may invest even more in reproduction to avoid fitness zero. Thus, we have discussed the possibility that differences in mating experience significantly affect the relationship between aging and reproductive investment. We corrected these sentences (Lines: 389-416).

L401-406: Frankly, I don’t think this is needed. I see no chance that resource availability might have influenced the results. Particularly as mating trials included food and were only 24h long, right?

Response: Removed this paragraph.

L417-419: This statement seems very generic. Why is it a problem that they have been in the lab so long? What differences might one expect from a natural population?

Response: Removed this paragraph.

References

Moiron, M., Winkler, L., Martin, O.Y. & Janicke, T. (2022). Sexual selection moderates heat stress response in males and females. Functional Ecology, 36, 3096–3106.

Spratt, E. C. (1980). Male homosexual behaviour and other factors influencing adult longevity in Tribolium castaneum (Herbst) and T. confusum Duval. Journal of Stored Products Research, 16(3–4), 109–114

Reviewer #2: I would like to thank the authors for the revision made, the manuscript is clearer now, and it is almost ready for publication.

There are some minor issues that shall be addressed. Indeed, it could be useful either to correct some grammar errors and correct some sentences. This shall be done either by another review to English or by having the manuscript reviewed by an English reviewer as suggested by the authors.

Response: Thank you for many useful comments to our manuscript. We corrected the manuscript as much as possible to accommodate the helpful comments from your comments. There is no doubt that our revised manuscript is better than before. We describe our response immediately after each your comment. We had an English proofreader correct the English in this manuscript. In this revised manuscript, our corrections are in red and corrections due to English proofreading are in blue. Please confirm.

Some species descriptors are lacking (for ex. lines 77, 79, 80, 116, 331).

Response: Sorry, we cannot understand this description. Could you please provide more details?

Sentences in lines 357-359 could be more elegantly written: "unclear and shall be examined in future studies".

Response: We corrected (Line: 373).

Some doubts about lines 370-372 - firstly you refer to a "strong dependence", then to a "small effect", is it possible to make it clearer?

Response: "Strong dependence" was used because there was a significant positive correlation between body size and paternity, but it was not necessary to use "strong," so "strong" was deleted from the sentence (Line: 381). Because the length of longevity may significantly influence our results, body size could have influenced present study if it was correlated with longevity. However, since there was no correlation between longevity and body size, we considered that effect of body size on our results may small.

Sometimes you refer only to paternity, but I believe that "success" shall be added after that word (ex. line 377).

Response: We added “success” after paternity throughout the revised manuscript.

Congratulations to the authors!

Response: Thank you so much.

Kentarou Matsumura

On behalf of all authors
